# Performance of Different Concrete Types Exposed to Elevated Temperatures: A Review

**DOI:** 10.3390/ma15145032

**Published:** 2022-07-20

**Authors:** Amjad Alhamad, Sherif Yehia, Éva Lublóy, Mohamed Elchalakani

**Affiliations:** 1 Civil Engineering Department, College of Engineering, American University of Sharjah, Sharjah P.O. Box 26666, United Arab Emirates; b00065547@alumni.aus.edu; 2 Department of Construction Materials and Engineering Geology, Faculty of Civil Engineering, Budapest University of Technology and Economics, H-1111 Budapest, Hungary; lubloy.eva@epito.bme.hu; 3 Department of Civil Engineering, School of Engineering, The University of Western Australia, Perth 6009, Australia; mohamed.elchalakani@uwa.edu.au

**Keywords:** elevated temperature, constituents, residual properties, spalling, cracking, fibers

## Abstract

Concrete is a heterogeneous material that consists of cement, aggregates, and water as basic constituents. Several cementitious materials and additives are added with different volumetric ratios to improve the strength and durability requirements of concrete. Consequently, performance of concrete when exposed to elevated temperature is greatly affected by the concrete type. Moreover, post-fire properties of concrete are influenced by the constituents of each concrete type. Heating rate, days of curing, type of curing, cooling method, and constituents of the mix are some of the factors that impact the post-fire behavior of concrete structures. In this paper, an extensive review was conducted and focused on the effect of concrete constituents on the overall behavior of concrete when exposed to elevated temperature. It was evident that utilizing fibers can improve the tensile capacity of concrete after exposure to higher temperatures. However, there is an increased risk of spalling due to the induced internal stresses. In addition, supplementary cementitious materials such as metakaolin and silica fume enhanced concrete strength, the latter proving to be the most effective. In terms of the heating process, it was clear that several constituents, such as silica fume or fly ash, that decrease absorption affect overall workability, increase the compressive strength of concrete, and can yield an increase in the strength of concrete at 200 °C. Most of the concrete types show a moderate and steady decrease in the strength up until 400 °C. However, the decrease is more rapid until the concrete reaches 800 °C or 1000 °C at which it spalls or cannot take any applied load. This review highlighted the need for more research and codes’ provisions to account for different types of concrete constituents and advanced construction materials technology.

## 1. Introduction

Fires around the world are more frequent than expected and occur due to several reasons. Civil engineering structures are always at risk of catching fire especially during the construction phase [1]. The use of wooden formwork that can easily catch fire makes this phase more vulnerable to fires. However, even when the construction is done, buildings are still not immune to fires. In fact, in the years 2000 and 2001, Taiwan had witnessed 5893 fires in residential occupancies [1]. Between 2008 and 2016, there were 5490 fires in Dubai. From these fires, 52.1% were related to residential units, commercial stores, industrial plants, construction sites, and government establishments [2]. Moreover, China as a country had a total of 132,497 fires in 2010 alone. This is a large number, especially when it is said that most of these fires took place in residential buildings killing 1205 people in the process and severely injuring 624 others [3]. In Australia, fires occurred in high-rise buildings due to the use of highly combustible lightweight thin aluminum alloy façades, even when the building’s management said that the building was compliant with the cladding standards of 2017. A government audit that was done after this disaster found that flammable cladding posed a threat to around 1400 buildings. Although it is true that some fires are intentional, most of them occur due to faults in electrical installations. This is very difficult to control because it is heavily influenced by human error. Furthermore, the number of fires is increasing annually due to increased use of electrical devices. Fires that take place around the world waste money, time, resources, and most importantly put human life at risk. Some buildings even collapse entirely when they catch fire. 

Within the last two decades there have been many advances in concrete technology and research. Fibers of different types, shapes, and sizes are being implemented into the concrete mix. In addition, modern concrete such as self-compacting (1980s) or self-healing (2000s) concrete are becoming points of interest when it comes to understanding how their constituents can affect their behavior when exposed to a fire or elevated temperature. 

One of the main topics under investigation is the effects of fire on concrete and how different constituents can improve the mechanical properties of concrete during and after fire. Since concrete has many different types, it is difficult for code developers and researchers to create a generalized equation that encompasses the fire and post-fire behavior of concrete. The current ACI and Eurocode codes [4,5] only discuss NSC and HSC under fire. However, they are still not comprehensive and do not provide equations that cover the wide range of concrete types, the different heating rates, or even the effect of different cooling types and how they affect the post-fire properties of concrete. This issue becomes even more complicated since it has been found that the constituents have a significant effect on the concrete’s properties under fire. An overview done in 2013 shows that steel fibers, aggregates, and various types of reinforcement all behave differently under elevated temperatures [6]. More specifically, steel fibers lead to a variety of results under fire depending on their type and volumetric ratios [7,8,9]. In addition, aggregates can change the post-fire properties of concrete depending on their type, size, and shape as well [10,11]. For instance, Diabase (also known as Dolerite) aggregates that are sort of eruptive show better results than dolomite or river aggregates [12]. In addition, it was found that using 20 mm aggregates decreased the risk of spalling significantly when compared to 7 mm or 14 mm [13]. This was due to the fact that larger aggregates provide a longer fracture process zone. This means it needs more kinetic energy to crack the concrete. Furthermore, it was shown that different types of stones such as limestone, quartz, and andesite behave differently with an increase in applied temperature. For example, limestone yields the lowest fineness modulus when compared to andesite or quartz. However, the most significant loss is present in quartz due a transformation phase at 573 °C that leads to a volumetric increase; this temperature induces cracks in the concrete, consequently weakening the concrete. On the other hand, andesite showed a slight increase in the fineness modulus of the crushed rock up until 600 °C [14]. In addition to aggregates, binders such as silica fume or metakaolin can also lead to certain improvements in the concrete behavior; nonetheless, silica fume proved to be better than metakaolin when it comes to performance during elevated temperature. Concrete with metakaolin has a very dense pore structure, which could trap the vapor inside the concrete leading to built-up pressure that can cause spalling [15]. Furthermore, these binders, aggregates, and fibers behave differently under certain heating rates and cooling types. 

Many research efforts indicated that there is a difference in the mechanical properties of concrete after being exposed to high temperatures based on the method of cooling [16,17,18]. There are mainly three types of cooling being studied in the literature. Water cooling, naturally cooling inside the oven, and naturally cooling outside the oven are conditions that are very important to compare since each one of them can create a change in the post-fire behavior of concrete. Most studies tend to subject the concrete specimens to natural cooling, which means that the specimens cool to room temperature inside or outside the oven without quenching or spraying with water. Natural cooling inside the furnace represents the scenarios in real life in which a fire is taking place but the sprinklers or fire extinguishers have not been used while the space is closed. Natural cooling outside the furnace represents a similar scenario except that air from outside the heating location can affect the temperature gradient inside the fire location. Moreover, quenching in water is representative of the application of water from sprinklers or fire fighters. This method of cooling could lead to injuries if the concrete specimens are not handled properly since the concrete is under a high risk of spalling. Most studies implement natural cooling inside or outside the furnace because researchers consider safety while conducting the experiments. Nevertheless, there are several studies that still study the effects of water cooling, but they are not as common as those that apply natural cooling. In general, there is a consensus in the literature on the idea that water cooling has a larger negative effect than air cooling. 

**Figure 1:**
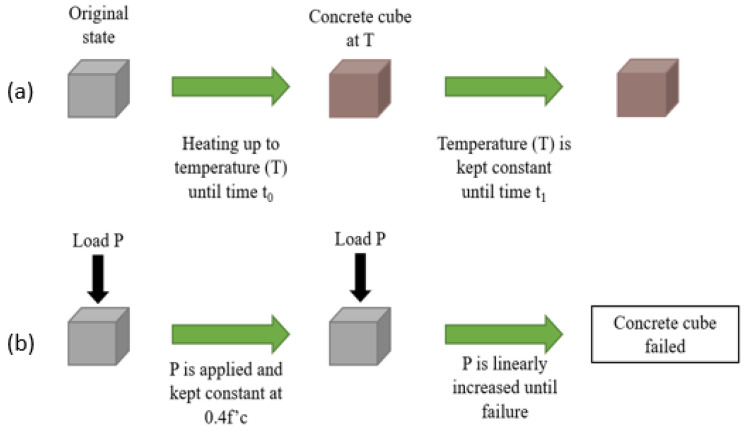
(**a**) Heating and (**b**) loading schemes of the stressed condition.

**Figure 2:**
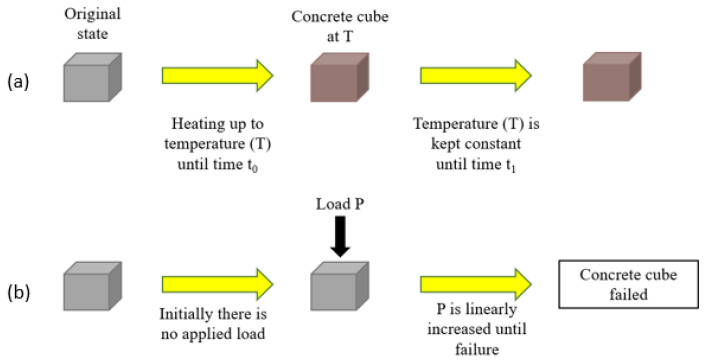
(**a**) Heating and (**b**) loading schemes of the unstressed condition.

**Figure 3:**
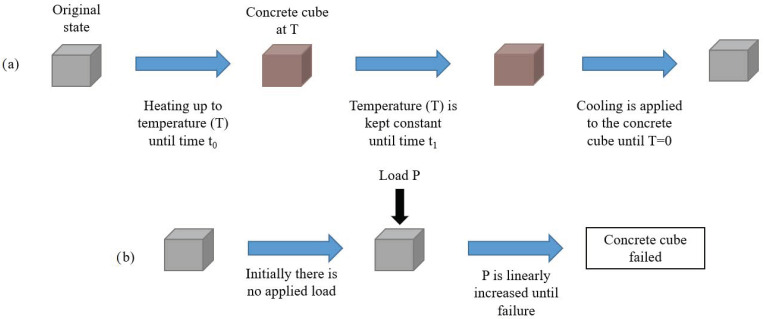
(**a**) Heating and (**b**) loading schemes of the residual condition.

Moreover, there are three types of testing regimes that are followed in the literature, namely “stressed”, “unstressed”, and “residual”. In the stressed method, a preload is applied to the concrete specimen and then maintained during the heating phase. Then, when the target temperature is reached, the temperature is sustained and the load is applied further until the concrete fails. This method, being the most representative of real life due to the preloading, is significantly less represented than the other two since it can be more difficult to conduct, as the load has to be constant while the sample is inside the furnace and then increased when the temperature is reached. This needs a certain setup and procedure that are not required for the other two regimes. With the unstressed experiment, there is no preloading of the concrete samples. The samples are placed directly in the oven and heated to a target temperature. Once the temperature is reached, the load is applied to the concrete until it reaches failure. This method is highlighted under the “no cooling” sections of this study. Lastly, in the residual test regime, the concrete specimen is heated with or without preloading. Then, when the target temperature is reached, the concrete is then cooled to ambient temperature before the load is applied until failure. This method is the most present in the literature since it is the easiest to conduct in the lab while at the same time highlighting the effects of cooling on concrete. These experiments are illustrated in Figure 1, Figure 2 and Figure 3.

The current review focuses on the effect of concrete constituents on the behavior of concrete exposed to elevated temperature and different cooling methods; therefore, the discussion and comparisons will be mainly directed towards concrete of the same type that follow the same cooling method. For example, studies on normal strength concrete that is cooled in air will be compared with others of its type and so on. This will make it easier to isolate the effect of the aggregates and will in turn having fewer variables. The comparison of the results is split into two or three parts depending on the cooling method. These methods are naturally cooling outside of the furnace, naturally cooling inside the furnace, and water cooling. This study will present and analyze the different equations from the codes and the literature regarding the mechanical properties of concrete. Other reviews on the subject of concrete exposed to elevated temperature effects [19,20,21], are more specific about one type of concrete or one constituent. For instance, such reviews discuss steel fibers, polypropylene fibers, or nanosilica and do not include clear comparisons and data on the effects of cooling methods. This paper provides more thorough and comprehensive discussion by covering different types of concrete which were tested under elevated temperatures and various cooling schemes. 

## 2. Response of Different Concrete Types Exposed to Elevated Temperature

It is important to note that the physical and chemical changes in concrete are essential to discuss when concrete is subjected to high temperatures. When the heating process is still in the early stage, the concrete undergoes a decrease in mass around 100 °C. This decrease is caused by evaporation of water from the macro pores. Decomposition of ettringite (3CaOAl_2_O_3_•3CaSO_4_•32H_2_O) takes place between a 50 °C and 110 °C [22,23]. Then, at 200 °C, more mass is lost and the concrete starts to either gain strength or lose based on the different constituents used in the mix. This is shown in the discussion presented later of the different concrete types. It is also clear that water content (water-cement ratio), type of cement, and age of concrete influence the amount of water that is evaporated as well as chemically bonded. After that, when the temperature ranges between 450 °C and 550 °C, there is decomposition of not carbonated portlandite (Ca (OH)_2_ → CaO + H_2_O↑). This process leads to an endothermic peak and consequently to further loss of mass [24]. In fact, dehydration of portlandite causes the most significant loss of strength in concrete [25]. When the heat is further increased, a certain quartz inversion takes place at 573 °C with a smaller endothermic peak. This quartz inversion creates a 5% to 7% volume increase [26] that leads to significant deterioration of concrete. At later stages of heating (600 °C to 1200 °C) the concrete has very little load bearing capacity. It is also important to note that at around 700 °C, Calcium-Silicate-Hydrate (CSH) compound decomposes leading to a significant loss of concrete strength [27]. In the following sections, the relative strength is considered to be the ratio of the strength at a specific temperature to the strength at room temperature. This ratio is used to compare the performance of concrete samples with different constituents under varies cooling conditions.

### 2.1. Normal Strength Concrete (NSC) 

NSC is the commonly used concrete type in the construction industry. NSC is moderate in terms of its features when compared to other types of concrete such as High Strength Concrete [28]. Its matrix is not as highly porous as lightweight concrete and not as dense as ultra-high-performance concrete. With a compressive strength of ranging from 20 MPa to 50 or 55 MPa (range is slightly different between different concrete codes) [28], NSC is used in different structural concrete applications, furthermore, its simple constituents make it cost effective than other concrete types that rely on the addition of different additives. However, NSC could be prepared with different aggregate types, binders in addition to cement. Pozzolanic Supplementary Materials are used extensively throughout the world. During the hydration of cement, Calcium Hydroxide is produced and reacts with pozzolanic materials. The amorphous silica present in the pozzolanic materials combines with calcium hydroxide and forms cementitious materials that act as the binder within the concrete matrix. Any Supplementary Materials (Silica Fume, Fly Ash, GGBS, etc.) having pozzolanic behaviour are capable of improving the durability of concrete and can also reduce the rate of heat liberated due to hydration, which is beneficial for mass concrete applications. 

Moreover, using pulverised fly ash (PFA) and slag in Portland cements (PC) can improve the concrete behavior at higher temperatures and allow it to sustain its mechanical properties. Furthermore, PCs blended with PFA and slag also exhibit a high resistance to spalling at high temperatures [28,29,30,31,32]. Several research initiatives are trying to replace cement with different types of binders such as silica fume or fly ash to improve the properties and durability requirements of concrete structures [31]. These initiatives also contribute to the efforts to reduce carbon (CO_2_) emissions. The use of more sustainable supplementary cementitious materials could reduce the CO_2_ emissions by 12% [33]. China alone produces 0.224 gigatons of CO_2_ from cement production [34]. Consequently, there is a clear need to explore the present alternatives to reduce cement and how the use of such alternatives can affect the properties of concrete under fire. Many studies were found in the literature that focused on post-fire properties of NSC. In addition, other studies that did not focus on NSC under fire still have a control mix that falls under the NSC category. Therefore, an overall comparison of the data found in the literature is presented. The discussion is divided to three categories depending on the cooling method used in each study. 

#### 2.1.1. Natural Cooling outside the Furnace

**Figure 4:**
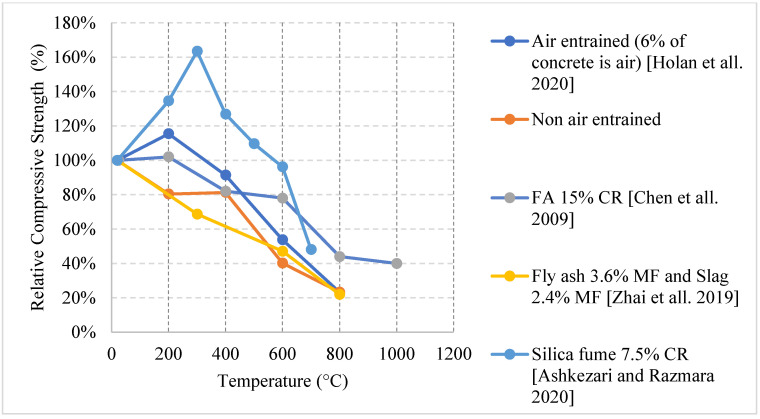
Effect of different constituents on the relative compressive strength of naturally cooled NSC. CR: Cement Replacement, MF: Mass Fraction [35,36,37,38].

**Figure 5:**
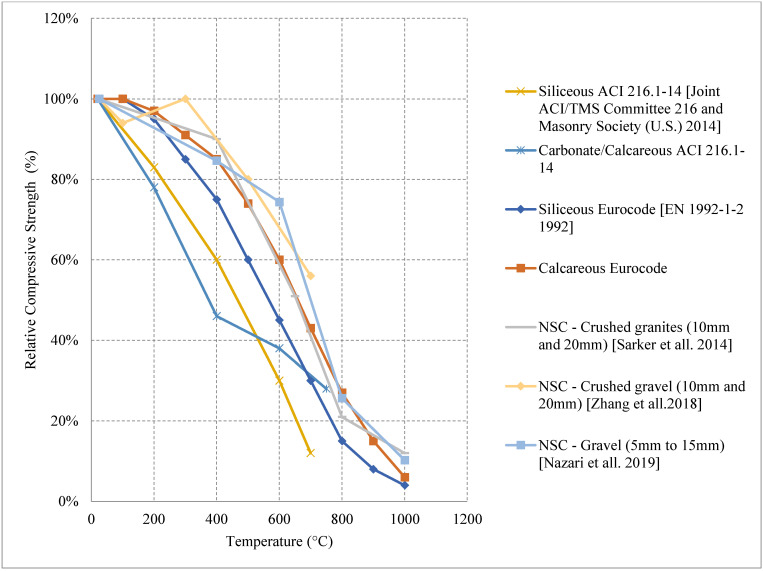
Effect of different constituents on the relative compressive strength of naturally cooled NSC [4,5,39,43,44].

Normal strength concrete (NSC) matrix can yield from different combinations of constituents, and changing their percentages can result in a variety of mixes with different microstructure, strength, cracking behavior, bond, and mass loss. Loss of compressive strength is a common behavior of NSC when exposed to high temperatures; however, in some cases, 20% or 60% increase in the compressive strength was reported, as shown in Figure 4 [35,36,37,38]. The increase is attributed to the extra C-S-H binder created by a delayed reaction of silica fume. A 7% weight replacement of cement with silica fume can create a 60% increase in strength at 300 °C and retains its original strength at 600 °C. This value is significant since most NSC samples would have lost more than 20% of their strength at 600 °C. In addition, other samples with 6% air-entraining agents (AEA) [35] showed about 20% increase which could be explained by the increased porosity created by the addition of the AEA. The difference between an air-entrained and a non-air-entrained sample is almost 50% at 200 °C [35]. The higher porosity allows the concrete to release some of the internal vapor pressure and eventually enhance the performance. However, increasing the porosity is not that effective after 400 °C, and the NSC will behave the same regardless of the porosity due to the large weight loss after 400 °C. Furthermore, NSC is sensitive to weight loss; a 5.7% loss in weight can result in about 20% drop in the compressive strength [39]. Another important parameter that could affect the post-fire behavior is the coefficient of thermal expansion. This coefficient depends on the aggregate type, proportion, and the water-cement ratio used in the mix [40]. Moreover, using siliceous aggregates can lead to a higher strength loss than concrete with calcareous ones where siliceous aggregates have a higher thermal conductivity than calcareous aggregates [41]. This allows the heat to flow smoothly into the concrete and reach deeper parts of the concrete section faster causing larger reduction in strength. In addition, quartz in the siliceous aggregates can undergo a phase transformation at around 570 °C, which can lead to a significant increase in volume, which in turn leads to internal cracks and more strength loss in the concrete. In fact, calcareous aggregates are predicted to maintain about 76% of their strength at 835 °C as opposed to siliceous aggregates that can maintain up to 66% [41]. Limited studies discussed the effect on flexural strength; however, it was found that silica fume is also capable of increasing the flexural strength at lower temperatures by almost 10% [42]. From Figure 5 [4,5,39,43,44], the residual data from the Eurocode [5] and the ACI 216-14 [4] are compared to other studies. It is clear that there is a variation in the results, the Eurocode is significantly less representative of the nature of different concrete types. The difference between the codes and different NSC mixes becomes more evident when comparing the data in Figure 5.

The use of nanomaterials as a replacement of cement affected the impact strength (resistance of sudden load or shock) of NSC after exposure to elevated temperature. This effect is influenced by the type and percentage of the nanomaterial used in the mixtures. The impact strength tends to increase at 250 °C when 10% of the cement is replaced with nano cement due to the extra gel it creates when reacting again at that temperature. The results vary and there is a difference in the percentage of impact energy retained by each percentage of each nanomaterial. The difference could sometimes be small but there are some exceptions. For instance, the 30% Nano metakaolin replacement lost all of its impact strength at 750 °C unlike the nano silica fume, nano fly ash, and nano cement [41]. This could be attributed to the fact that metakaolin is produced by the calcination of kaolinitic clay, which is less resistant to heat than the silicon that makes the silica fume or the pulverized coal that makes the fly ash. 

#### 2.1.2. Natural Cooling inside the Furnace

**Figure 6:**
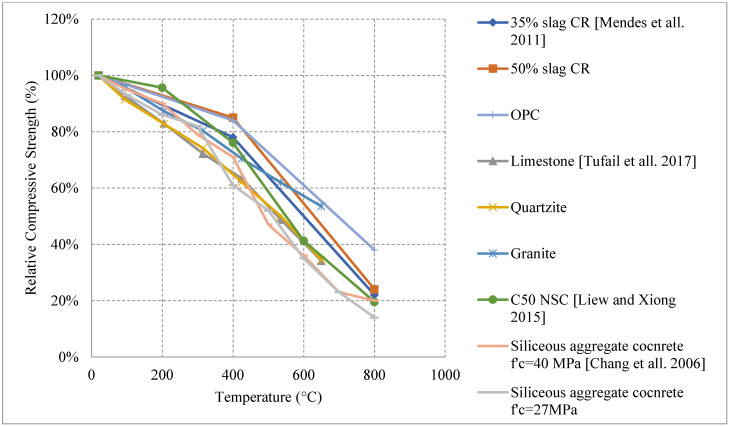
Effect of different constituents on the relative compressive strength of furnace cooled NSC. CR: Cement Replacement [45,46,47,48].

For this type of cooling, the concrete is expected to yield similar results to natural cooling outside of the furnace since there is no shock (sudden change in temperature) such as in water cooling. However, the rate of cooling becomes slower since the concrete is not exposed to air. All the results shown in Figure 6 [45,46,47,48] follow a decreasing trend in terms of strength. It is important to note that no silica fume, fly ash, or metakaolin are used by any of these studies. This might be one reason that there is no recovery or increase of strength. In addition, slag with different percentages has no positive impact on the residual properties of NSC [44]. However, changing its percentage can yield different results, for instance, changing the percentage of slag from 35% to 50% within the concrete will have slightly more resistance to fire. Moreover, it was emphasized that the type of coarse aggregate used can have an impact on the behavior of NSC. Concrete with either quartzite or limestone aggregates shows similar behavior and keeps only 35% of its strength at 650 °C. Nonetheless, at the same elevated temperature using granite aggregates proved to retain about 54% of the concrete’s strength. In addition, the granite concrete had less degradation at every temperature. These results could be explained by the original high strength of the granite and crystalline nature, which allow the aggregates to be more resistant to the elevated heat. In addition, due to its higher thermal resistivity, granite aggregates yield a higher modulus of elasticity the other quartzite and limestone. Test results by [47] showed that modulus of elasticity of specimen prepared with granite was reduced by about 48% at 650 °C while these prepared by limestone or quartzite lost around 70% of the modulus values. Another phenomenon is that limestone has the lowest thermal expansion but still losses more strength due to the partial decomposition of calcite, the phase transition of quartz, and the internal cracks that develop with an increase in temperature. The change in the behavior relies on the type of curing as well. In [47], a comprehensive discussion on the properties of fresh concrete, hardened concrete, admixtures, and temperature effects on concrete is presented. The temperature effects are not only significant during the heating process, but steam curing could affect the mechanical properties of concrete significantly based on the duration and the temperature of the environment [49]. 

#### 2.1.3. Water Cooling 

**Figure 7:**
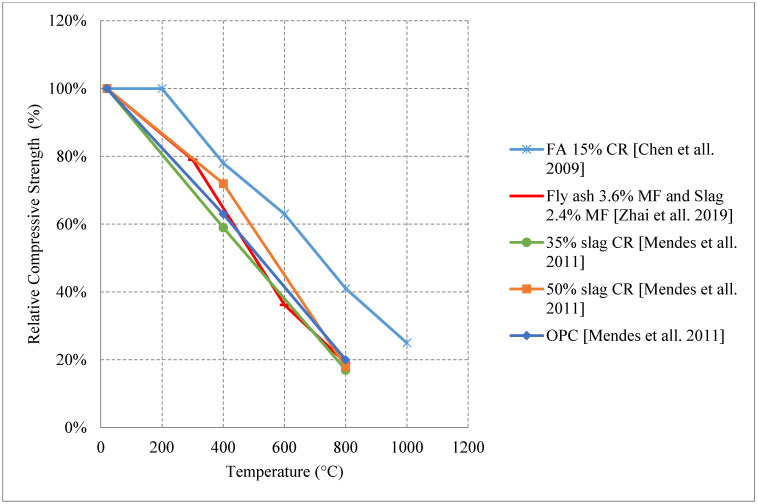
Effect of different constituents on the relative compressive strength of water cooled NSC. CR: Cement Replacement, MF: Mass Fraction [36,37,45].

Unlike naturally cooled concrete, water cooled concrete shows no increase in strength between 200 °C and 400 °C. In fact, most of the tested samples retain around 50% of their compressive strength followed by a continuous drop in strength at temperatures above 400 °C [36,37,45,50,51]. Moreover, the drop-in strength is more rapid due to several reasons. This rapid loss of compressive strength could be attributed to the thermal shock created when water cooling changes the temperature instantly. This creates a temperature difference between the surface and the inner core of the specimens and forms additional internal stresses. It is reported that the use of silica fume did not yield a significant increase in strength similar to specimens naturally cooled. Metakaolin is another one useful binder that is not studied under water cooling in the literature. Using 50% slag as binder replacement would improve the properties of NSC when water cooled, as shown in Figure 7 [36,37,45]. Using slag reduces the permeability of concrete, and its high durability can reduce the cracks under high temperatures and increase the modulus of elasticity of concrete yielding a better performance; however, its effect is insignificant. Instead, using 15% fly ash yields the best performance overall. It also provides enough durability that can slow down the flow of heat into the deeper parts of the cross section. Nonetheless, the use of a lower percentage of fly ash can reduce the internal stresses within the concrete since it improves the porosity. Moreover, more fly ash would increase shrinkage and eventually deteriorate the concrete at higher temperatures [36,37]. Fly ash can be efficient since it has a low heat of hydration, which prevents thermal cracking. In terms of splitting tensile strength, fly ash could be considered inefficient due to the increased brittle nature of concrete leading to the creation of more cracks. In general, NSC performs much worse when water cooled. Since this type of cooling has the most negative effect on concrete, the use of different constituents should be analyzed thoroughly in order to improve the post-fire performance. 

Table 1 [35,36,37,38,43,44,45,46,47,48,51] summarizes some of the important parameters found in the literature. Specimens in most of the studies were tested after 28 days and exposed to similar heating rates. The most significant differences between these studies are aggregate types, addition of different binders, and admixtures. Given the significant difference in the constituents and their volumetric ratios in each study, it is evident that there is a large variety of unique mixes that need to be evaluated to truly understand the effects of fire on concrete prepared with different constituents.

### 2.2. Lightweight Concrete (LWC) 

LWC is common within the construction industry because there are many design scenarios where LWC can be more cost effective than NSC. Especially when designing high-rise buildings and floating structures like pontoons and floating bridges [52,53,54,55], the lower dead weight of the LWC can save large sums of money. The data presented in this section is based on studies that are directly investigating the properties of LWC exposed to elevated temperature. There is a large variation between these studies since the type of curing, the absorption of the aggregates, the abrasion resistance, and crushing values of the aggregates can make a great difference in the post-fire properties. The discussion on LWC is limited to data presented by journal articles since the codes do not present any data or equations that can summarize the post-fire properties of this type of concrete. However, the ACI code [4] does provide data regarding semi-lightweight concrete, but it is still not representative of the wide range of results found in the literature. Although LWC is a common topic among researchers, its thermal properties are not when compared to other types of concrete such as FRC, NSC, or HSC. LWC concrete also proves to have a lower thermal conductivity due to its highly porous nature [56]. Moreover, the high porosity of the LWC can significantly affect its post-fire residual properties. There is in fact an inversely linear relation between the compressive strength and the porosity at room temperature [57]. Therefore, these pores make the LWC behave much differently than other types of concrete and can play a big role in the loss of strength. However, it is still not clear whether it is better than other types of concrete in terms of residual strength. LWC suffers more from spalling than some of the other concrete types due to its high-water absorption. Its aggregates absorb a large amount of water which evaporates at elevated temperatures and significantly weakens the concrete matrix. This is mainly due to the lack of studies on the residual strength of LWC. There are even less studies targeting the post-fire effects of LWC constituents. Since LWC is commonly used today, it is important to thoroughly study its post-fire properties and how different constituents can improve these properties in case of exposure to elevated temperatures or fire. 

Since 2012, researchers have tried improving the properties of LWC using different constituents [54,55,56,57,58,59]. For instance, recycled glass was found to yield good strength values while reducing the density of LWC when mixed with metakaolin [58]. It is clear from the literature that a lower density is preferable when trying to resist spalling. Moreover, when coarse aggregates were replaced by activated carbon that is produced from oil palm kernel shell (OPKS), the OPKS activated carbon lightweight concrete was able to achieve a strength of 50 MPa [59]. Given this efficient result, the fire properties of LWC will be unique since the carbon itself has high water absorption and increases the thermal conductivity. Last, a study implemented the use of micro fly ash cenoshperes (FACs) and found that it could reduce both the density and the thermal conductivity of LWC. In addition, because of their small size and strong shell, the FACs are able to slow crack propagation [60]. All these new ideas require studying under fire in order to truly understand their effectiveness. 

Studying the residual properties involves applying different cooling methods to the samples after exposure to different heating rates. Studies found in the literature on the residual properties are limited and cannot be directly compared since they either use a different cooling method or add fibers. The results will be generally discussed.

#### Literature Results Performance of LWC Exposed to Different Cooling Conditions

**Figure 8:**
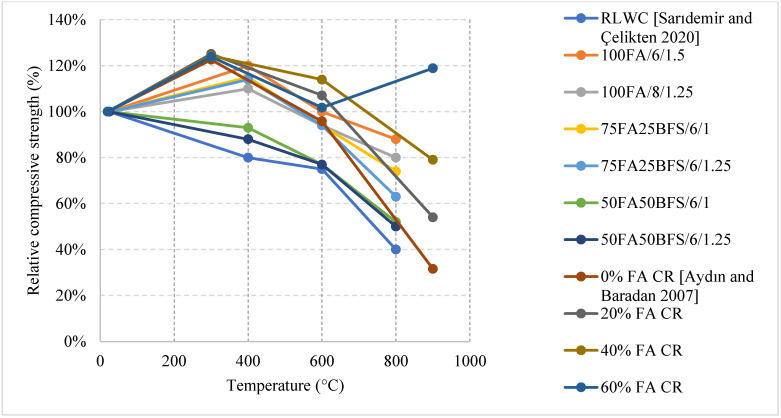
Relative compressive strength of LWC cooled at 0.5 °C/min. R: Reference, FA: Fly Ash, BFS: Basalt Furnace Slag, 100: 100% Cement replacement (CR), 75: 75% CR, 25: 25% CR [61,62].

After an investigation of the behavior of LWC under fire, it was found that LWC could easily undergo an increase at 300 °C. This happens when free water evaporates forcing the cement gel layers to move closer together and strengthening the overall cement paste. It could also be due to the improved hydration and increase of C-S-H content. Moreover, many studies implement fly ash into the LWC mix in order to replace cement. When tested under fire, LWC with more than 20% fly ash is always better for maintaining the concrete’s compressive strength when compared to 0% fly ash concrete [61]. As shown in Figure 8 [61,62], when fly ash replaces 60% of cement, the residual compressive strength at 900 °C is about 119%. This is relatively large since at 900 °C many LWC specimens would have completely lost their strength because of the deteriorating matrix. However, it was attributed to the enhancement of the bond at the interfacial transition zone as well as the conversion of the LWC’s microstructure from hydraulic to ceramic at that temperature [62]. The fly ash certainly has many positive effects on LWC. Nonetheless, when it comes to flexural strength, concrete becomes more brittle with an increase in fly ash content, which makes it weaker in tension. 

**Figure 9:**
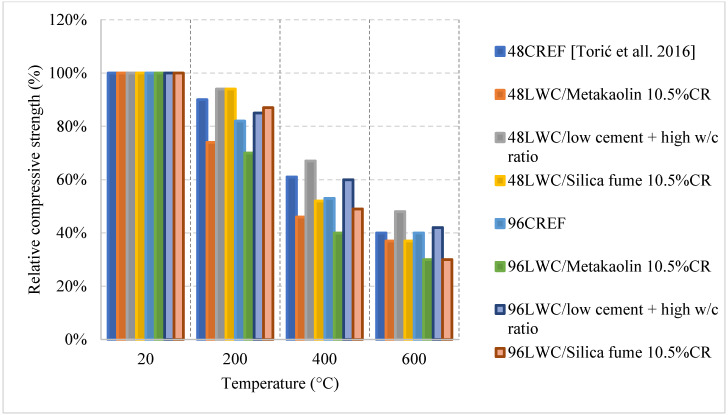
Relative compressive strength of LWC cooled at 1 °C/min. 48LWC: Specimens that are tested after 48 h of cooling, 96LWC: Specimens that are tested after 96 h of cooling, CR: Cement replacement [63].

Different results were reported by several studies, and the differences are not only due to the cooling or heating methods but due to the constituents used in the mix. For instance, it is shown in Figure 9 [63] how mixes with a low cement content and a higher w/c ratio can yield better post-fire performance. This could be explained by the high internal stresses induced by the volume expansion in the cement paste when specimens had high cement content. Most mixes tend to show a direct loss of strength, but the one with low cement content yielded a slower loss due to its lower moisture absorption and lower cement rehydration. In general, it is expected that the rehydration of cement yields an increase in strength; however, in this case it is more harmful than useful. Moreover, the addition of other binders such as fly ash and GGBS were investigated and proved to be very beneficial in terms of the post-fire properties. As shown in Figure 8, the use of fly ash significantly improved the residual compressive strength. The concrete that used fly ash witnessed a strength increase at lower temperatures. This is due to the reaction of the excess fly ash that can create additional gel and in turn improve the bond characteristics of the LWC. LWC that incorporated fly ash retained about 80% of its compressive strength. However, using fly ash with Basalt Furnace Slag (BFS) would show worse results when compared to just using fly ash. The increased BFS amount led to an increase in the calcium-alumino silicate-hydrate (C-A-S-H) gel content. Since (C-A-S-H) is more vulnerable to degradation than sodium-alumino silicate-hydrate (N-A-S-H) gel, the compressive strength was lower. 

**Figure 10:**
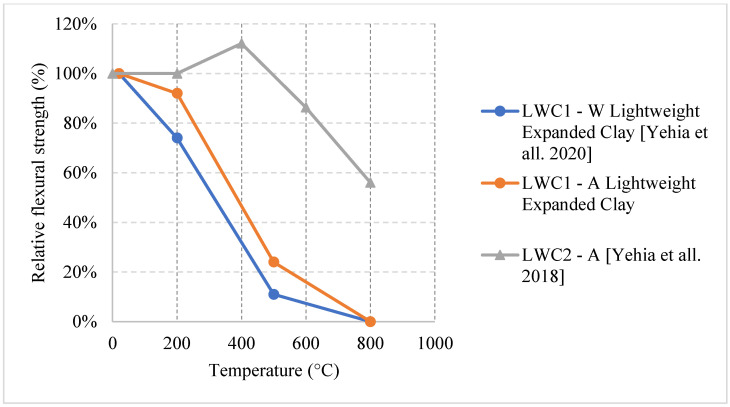
Relative flexural strength of naturally/water cooled LWC. W: Water cooled, A: Air cooled [64,65].

**Figure 11:**
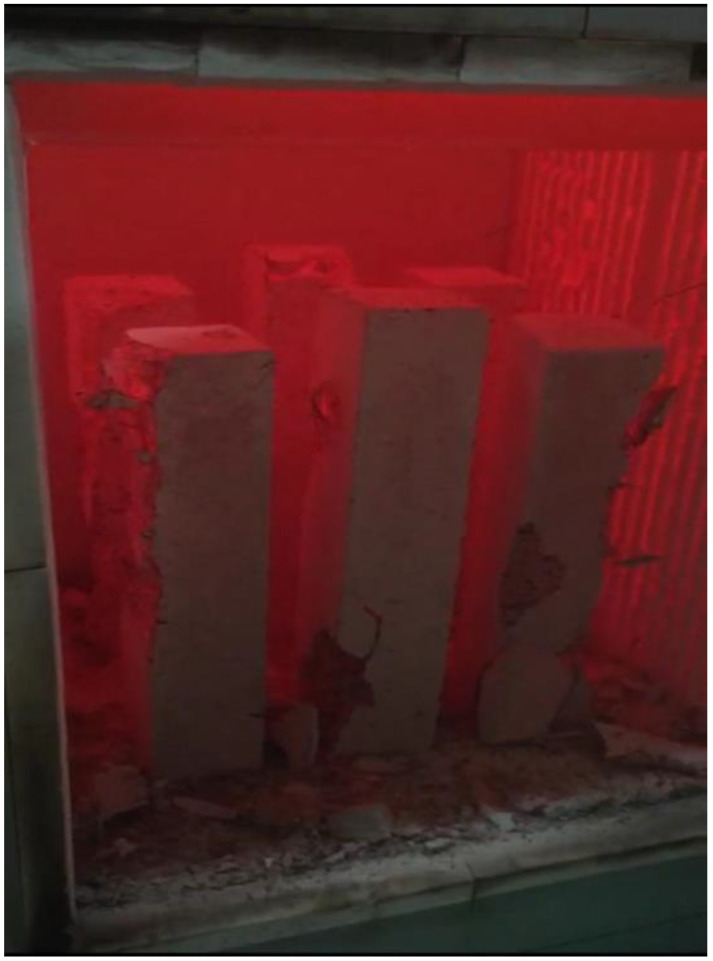
Samples that spalled inside the oven.

**Figure 12:**
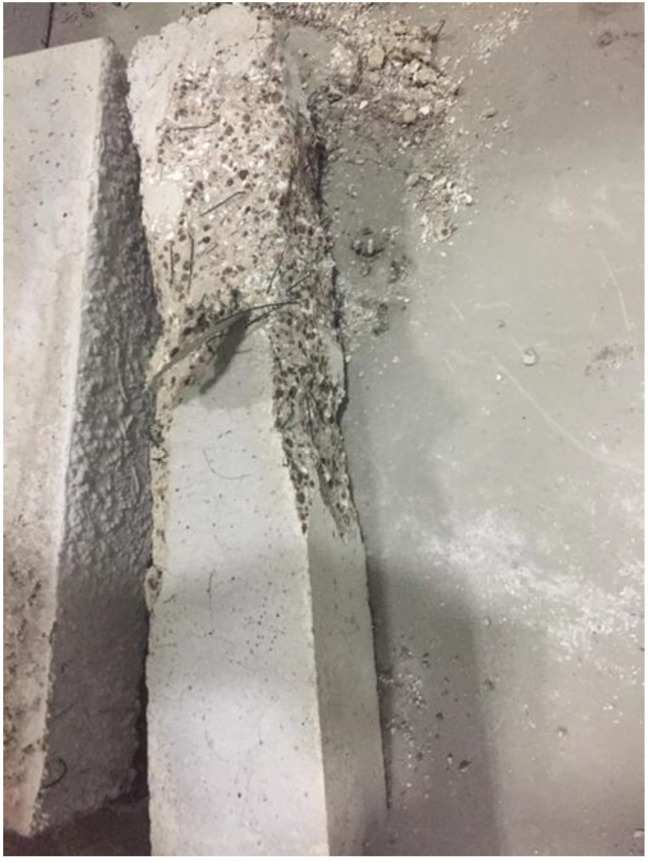
Deteriorated surface of 500 °C sample after water cooling.

Several research efforts on LWC by the authors have shown different results when using normal and water cooling [64,65]. Figure 10 [64,65] shows results from two different mixes of LWC under different cooling methods. During the heating phase, it was noticed that placing the samples close to each other changed the temperature distribution and the gradient within the concrete. Due to that, many samples (as shown in Figure 11) have spalled and the heating phase was repeated adding only few samples at a time inside the furnace [64]. In addition, the type of cooling had a clear effect as the thermal shock due to the applied water was able to reduce residual flexural strength of LWC. There was a slight increase in compressive strength at 400 °C due to the addition of C-S-H gel as cement rehydrates. Then, the strength drops almost linearly. Moreover, during the cooling phase, the LWC samples shown some surface deterioration as shown in Figure 12. This happened due to the thermal shock caused by the application of water. The samples were already heated to 500 °C when this took place. This means that the surface of the LWC was already fragile due to the high level of heat. 

As shown in Table 2 [61,62,63,64,65], all the studies on LWC used a different set of coarse aggregates, fine aggregates, and w/c ratios. Even the binders and the heating rates used were considerably different. This leads to many more questions regarding this type of concrete and its post-fire residual properties. 

### 2.3. High Strength Concrete (HSC)

HSC is another type of concrete that mostly uses additional binding materials such as silica fume, metakaolin, and GGBS. It has an overall better strength, durability, and a higher modulus of elasticity than NSC. HSC relies on using a lower w/c ratio and making the matrix denser by increasing the volume of cementitious materials and fine aggregates. When compared to NSC under fire, HSC yields better results at the early stages of heating [66]. In a number of studies, it could succeed NSC at every stage of heating [67,68]. However, its behavior is still unclear and could vary significantly under fire depending on the heating rate, cooling method, and the constituents used in the mixture [69]. Moreover, it is more susceptible to explosive spalling than NSC at higher temperatures due to its low permeability and the overall dense matrix, which tends to trap the evaporating water, consequently, increases the internal stresses. It was found that this high-water vapor pressure could reach up to 8 MPa [70]. This pressure is considered high given the fact that some HSC beams have a tensile strength of 5 MPa [71]. There are studies in the literature that search for ways to minimize the risk of spalling. It was found that using limestone aggregates and less silica fume content (less than 10% per weight) could reduce the risk of spalling [72]. This is mainly due to the fact that limestone aggregates have increased durability and can reduce shrinkage when compared to other aggregates. In addition, reducing silica fume increases the permeability of the HSC matrix and allows for more pressure relief. Another method that can reduce spalling was the implementation of Polypropylene (PP) fibers in the HSC mix. PP fibers melt at early temperatures and can leave behind channels within the concrete matrix that can increase permeability and allow for vapor movement [73]. In general, resistance of HSC to fire is still being investigated by many researchers. It is very important to study all the parameters that contribute to its post-fire behavior. Especially when spalling is an issue, constituents are very important in order to resist its negative effects and provide a safe structure that does not explode under fire. 

#### 2.3.1. Natural Cooling outside the Furnace

**Figure 13:**
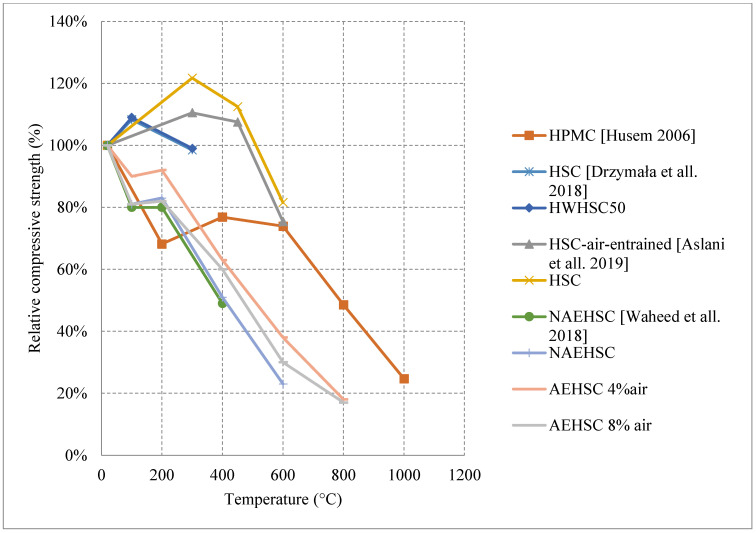
Relative compressive strength of naturally cooled HSC with different constituents. HPMC: High Performance Micro Concrete, HWHSC: Heavy Weight High Strength Concrete, NAEHSC: Non-Air-Entrained High Strength Concrete, AEHSC: Air-Entrained High Strength Concrete [67,74,75,76].

**Figure 14:**
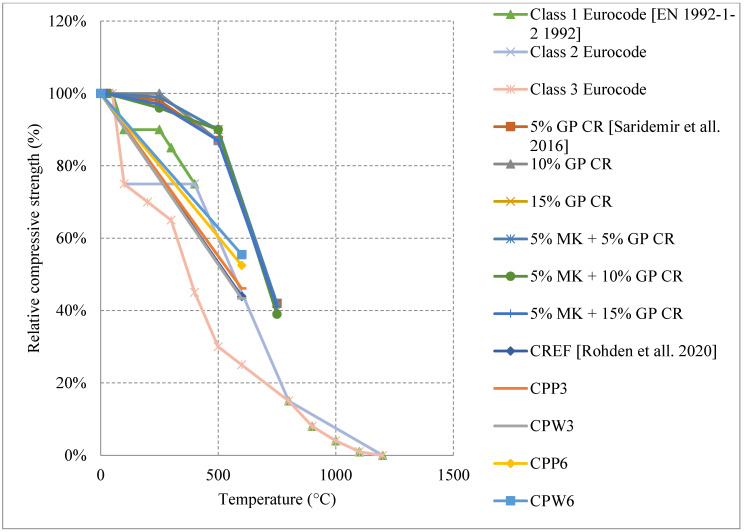
Relative compressive strength of naturally cooled HSC with different constituents. MK: Metakaolin, GP: Ground Pumice, CR: Cement Replacement, CPP3: Concrete with 3 kg/m^3^ Polypropylene fibers, CPP6: Concrete with 6 kg/m^3^ Polypropylene fibers, CPW3: Concrete with 3 kg/m^3^ Plastic Waste, CPW6: Concrete with 6 kg/m^3^ Plastic Waste [5,68,77].

Research on HSC has shown diverse results in some sections and very limited results in others. HSC samples that were tested after being naturally cooled showed very unique results when changing their constituents. It was observed that HSC can be very weak and vulnerable to spalling when exposed to temperatures up to 300 °C. This is also true for those HSC samples that contain heavyweight magnetite as a coarse aggregate with 50% replacement ratio. The low permeability of HSC increases the built-up pressure within the concrete leading to very weak results, as shown in Figure 13 [67,74,75,76]. In addition, samples with 75% and 100% magnetite replacement ratio spalled instantly due to the exceptionally large stresses that formed inside the very dense concrete specimen. On the contrary, air entertaining admixtures (AEA) when added to concrete, specimens could sustain around 80% of their strength at 600 °C [75]. The microscopic bubbles created inside the concrete, reduce thermal conductivity as well as the pressure that builds up in the concrete when it is heated. However, AEA can be significantly advantageous to use only in certain cases. Observing the behavior of different HSC samples found that a mixture of both silica fume and AEA was better than the single use of silica fume and worse than the single use of AEA. This could be mainly due to the added pressure that forms inside the concrete due to the expansion of silica fume. In some cases, the concrete spalled at 500 °C due to this expansion. Furthermore, HSC was found to perform better when exposed to elevated temperature when coarse aggregates are taken out of the mix. In fact, this High-Performance Micro Concrete (HPMC) could retain 50% of its compressive strength at 800 °C [67]. The lack of coarse aggregates helps the concrete reduce some of the internal stresses that form when the concrete is heated. Although initially it loses its strength very quickly, at 200 °C the concrete starts to gain back some strength before falling back again [67]. Regaining compressive strength is attributed to the evaporation of free water and the removal of the water of crystallization from the cement paste. Other HSC specimens have shown spikes in strength at the earlier stages of heating [74,75]. This can be due to the additional C-S-H created by the common reaction of cement. This reaction improves the bond between constituents improving performance of the HSC resisting higher temperatures. Additionally, results presented in Figure 14 [5,68,77] show that adding metakaolin or ground pumice proved to yield the best results [68]. In fact, it could maintain at least 70% of its strength at 500 °C [68]. Although metakaolin closes the pores within the HSC matrix making it denser and more susceptible to spalling, it could also mitigate expansion due to Alkali-Silica reaction and reduce the pressures that form within HSC [68]. This might be the reason that it gives HSC durability up to 500 °C. After that, many micro cracks start to form inside the concrete and the strength gradually starts dropping. These results when compared with the Eurocode show a great difference. The Eurocode provides data regarding three different classes of HSC. Concrete that is C 55/67 and C 60/75 is class 1, class 2 is concrete that is C 70/85 and C80/95, and class 3 is C90/105. The data shown in Figure 13 is clearly not representative since they are significantly different from the data in Figure 14. The large variety in the results found in the literature is evidence that different concrete constituents can affect the post-fire residual properties of concrete; therefore, the extent of these results should be investigated thoroughly. Other fibers such as PP fibers also improved the overall post-fire strength of concrete in both flexure and compression. 

**Figure 15:**
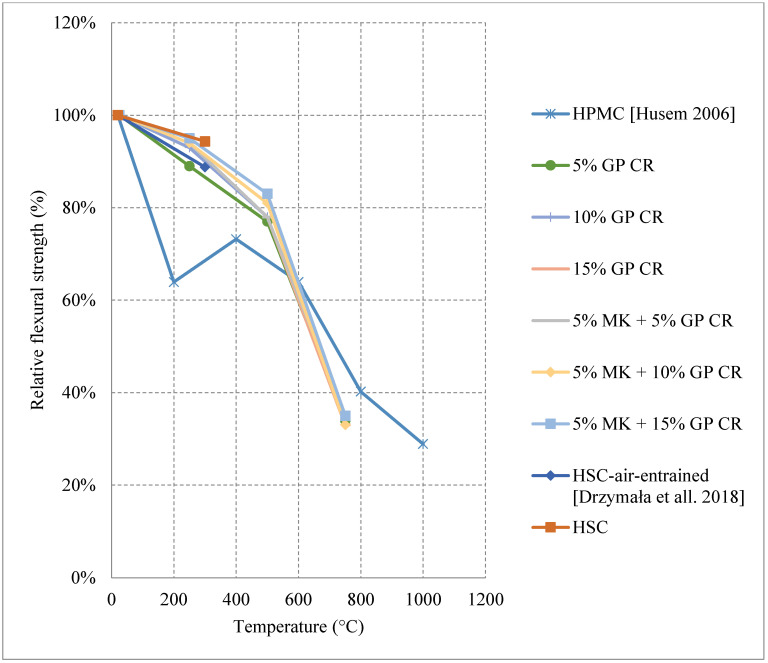
Relative flexural strength of naturally cooled HSC with different constituents. HPMC: Micro-High-Performance Concrete, MK: Metakaolin, GP: Ground Pumice [67,74].

**Figure 16:**
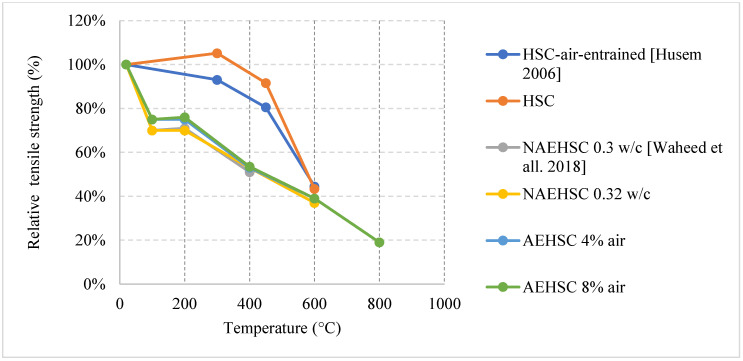
Relative tensile strength of naturally cooled HSC with different constituents. NAEHSC: Non-Air-Entrained High Strength Concrete, AEHSC: Air-Entrained High Strength Concrete [67,76].

**Figure 17:**
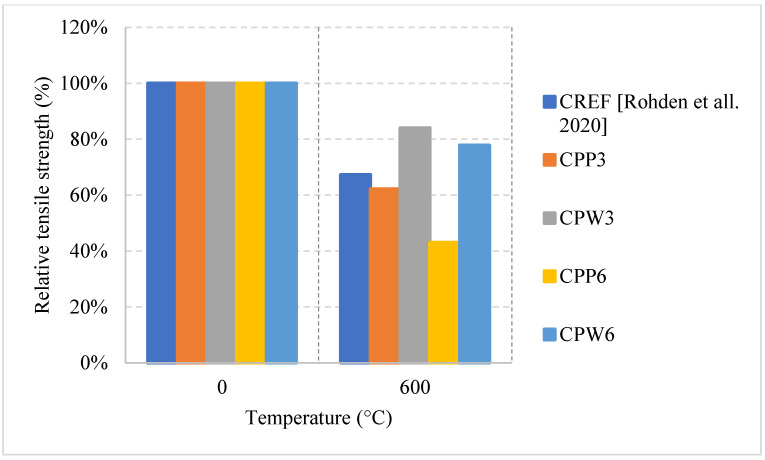
Relative tensile strength of naturally cooled HSC. CPP3: Concrete with 3 kg/m^3^ Polypropylene fibers, CPP6: Concrete with 6 kg/m^3^ Polypropylene fibers, CPW3: Concrete with 3 kg/m^3^ Plastic Waste, CPW6: Concrete with 6 kg/m^3^ Plastic Waste [77].

In terms of residual flexural or tensile strength of HSC, the use of Metakaolin or ground pumice proved to be the best at higher temperatures up to 600 °C, as shown in Figure 15 [67,74]. On the contrary to silica fume or fly ash, both of these constituents add significant resistance to fire. While metakaolin is able to increase the durability of HSC, the light nature of the ground pumice reduces the pressures that form within the concrete when it is subjected to high temperatures [67]. In fact, HSC samples with silica fume and regular coarse aggregates yield a drop in the tensile strength and some of them spall at very low temperatures. This could be attributed to the increased density and brittleness that are caused by an increase in silica fume content. When concrete becomes more brittle, it will naturally have a weaker flexural strength especially at higher temperatures when micro cracks start to form. It could also be due to the added pressures as a result of the expansion caused by the silica fume. HSC is better at conserving its strength when it contains constituents that relieve some of the internal built-up stresses that form when it is subjected to elevated temperatures. Moreover, the percentage of AEA used significantly affected the behavior, as shown in Figure 16 [67,76]. However, some samples that contained a mix of both AEA and silica fume were better than those with only silica fume [76]. This is mainly because AEA reduces the built-up stresses inside the concrete. In fact, AEA does not always yield better results; the use of AEA proved to be worse when compared to regular reference HSC that does not have any additional binders [74]. This was also the case when discussing compressive strength. It could be explained by the naturally weaker matrix created by the air voids. In general, constituents such as ground pumice, AEA, and metakaolin seem to help the concrete preserve its tensile strength under fire. Furthermore, High Performance Micro Concrete (HPMC) which relies only on fine aggregates could retain 30% of its tensile strength at 1000 °C after being subjected to cooling [67]. In addition, Figure 17 [77] shows the behavior of HSC after incorporating plastic waste. It was found that they could be better than using PP fibers as long as they are used as 3 kg/m^3^ or 6 kg/m^3^ quantities. However, the extent of these effects is still unclear. In addition, the current concrete codes, [4,5], are not comprehensive, as they do not consider effects of all the different constituents when discussing the mechanical properties of concrete. This further emphasizes the need for more studies to be done on the effect of these materials. A comparison between Figure 14 and Figure 17 shows that at 600 °C when the PP fiber content was 3 kg/m^3^ and that the relative tensile strength was larger than the relative compressive strength. However, when using 6 kg/m^3^, the opposite effect could take place. This is mainly due to the increased pore content that is created at higher temperatures by the deteriorating PP fibers. In addition, the vapor pressure network created by the continuous channels of PP fibers could damage the concrete matrix [77]. Other types of constituents such as plastic waste could be better as they create a discontinuity in the vapor network and in turn yield better results. Plastic waste also proved to reduce heat-induced concrete spalling due to their lower thermal conductivity; therefore, it was able to yield a better tensile strength at 600 °C than PP fibers [77]. Surely, HSC can be better than NSC in terms of its post-fire properties; however, it all depends on the type of constituents used and their amounts.

#### 2.3.2. Natural Cooling inside of the Furnace 

**Figure 18:**
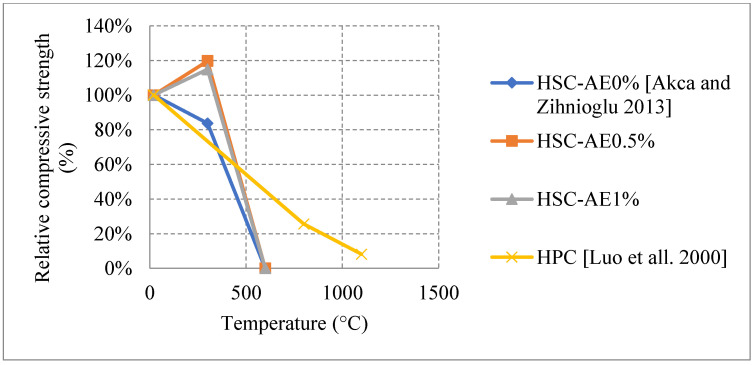
Relative compressive strength of furnace cooled HSC with different constituents. AE: Air Entrained [78,79].

When it comes to natural cooling inside the furnace, the studies in the literature made it clear that there are differences in the behavior of HSC due to the use of different materials. Such differences are shown in Figure 18 [78,79]. As discussed before, the use of AEA allows the concrete to gain strength between room temperature and 300–400 °C [78], which could be explained by the increase of the air voids inside the HSC, the decrease of the matrix density, and the reduction of thermal conductivity due to the addition of AEA. Subsequently, the concrete becomes less brittle and is able to take a higher static load when heated to 300 °C. The formation of additional C-S-H could also be a contributing factor to the strength increase. On the other hand, samples with no AEA tend to lose strength directly when heated. The HSC’s brittleness, its dense matrix, and the increased stress that forms within it will counter any effect that might allow for an increase in strength. Although the presence of AEA will allow for a strength increase at lower temperatures, the concrete faces a spalling issue at 600 °C [78]. This is due to the fact that water vapor cannot escape the created air voids and largely increases the internal pressures at high temperatures. 

#### 2.3.3. Water Cooling

**Figure 19:**
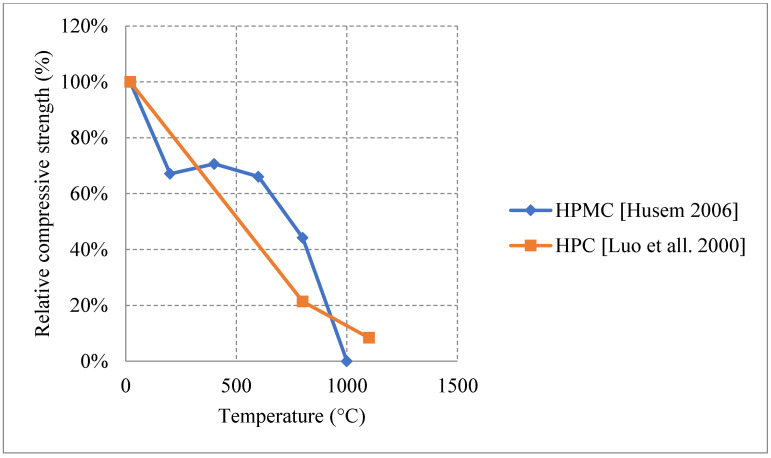
Relative compressive strength of water-cooled HSC. HPMC: High-Performance Micro Concrete [67,79].

Two studies indicated that there is a direct decrease in the compressive strength of HSC due to the negative effects that the thermal shock causes [67,79]. However, some samples of HSC showed a slight increase in strength after 200 °C [67]. This strength gain makes a significant difference as seen in Figure 19 [67,79]. The removal of the water of crystallization from the cement paste could explain such phenomena. It is evident that there is more strength gain in the naturally cooled specimens than those cooled in water since the added water reduces the crystallization process. This increase is not present when using a mix of fly ash and silica fume [79]. Mainly, the negative effects of the thermal shock along with the expansion of silica fume do not allow for any increase in the strength. However, for HSC with no coarse aggregates, the concrete can gain back some of its strength due to the lower internal pressures as a result of less dense matrix. Spalling is a main issue in water cooled HSC; many specimens spall because of the thermal shock, in addition to the increasing pressures created by water vapor and the expansion of silica fume. The stress difference between the outer surface and the inner matrix of the concrete can lead to spalling. Nonetheless, there are inconsistencies in the spalling problem that need to be investigated. Therefore, there is a need to investigate the constituents that are best at resisting load after water cooling since studies on this topic are scarce. 

Table 3 [67,68,74,75,76,77,78,79] summarizes different mixtures that were used in each of the studies included in the table. Since most of them use similar heating rates, curing days, w/c ratios, and cement types, the differences in behavior mainly come from the use of different cooling methods as well as different binders, admixtures, and aggregates. 

### 2.4. Fiber Reinforced Concrete (FRC)

Discrete fibers from different materials and aspect ratios (length/diameter) with various volumetric ratios are dispersed separately or together (hybrid) in the concrete matrix to improve mechanical properties and durability of concrete structures. In addition, they can be used to enhance the post-fire properties. Different types of fibers can elongate in the early stages of heating improving crack control and enhancing the strength. However, at later stages, spalling and cracking are very prominent issues due to the excessive stresses created by the elongated fibers and the lack of concrete tensile strength to resist this elongation. It is important to note that American and European codes [4,5] have not discussed the performance of different types of concrete and the extent of the use of different fiber percentages. The variation requires a lot of data to be able to derive equations that can represent the true nature of FRC. In addition, several studies found in the literature; however, they are not very representative of the overall behavior of FRC since there are many variables such as fiber types, aspect ratios, percentage, different constituents, and aggregates mixed with these fibers. Each unique combination of such variables yields different results when the concrete exposed to elevated temperature. Moreover, given the unique effect of water cooling, there is a need for more studies done around it since its combination with all the previously stated variables will exhibit a unique behavior. It is important to note that the behavior of cylinders or cubes under elevated temperature behave differently than panels. This is because of the temperature gradient within the concrete panel itself. Although it is tough for researchers to test panels under fire due to the sample size and the furnace size, it was shown in [80] that panels with 10% recycled rubber faced spalling at 7 min into the heating process. While the panels that used 0.5% or 1.5% steel fibers showed no sign of spalling. When compared to the results from the literature, it is clear that there is a difference in the behavior since steel fibers are known to increase the risk of spalling in cylinders or cubes. 

#### 2.4.1. Natural Cooling outside the Furnace

FRC specimens containing only Polypropylene (PP) or cellulose fibers reduced the tendency to spalling. The resistance to spalling was present in FRC when implementing hybrid fibers. This could be attributed to the relatively low melting point of the PP fiber. When the PP fiber decompose before 500 °C, the concrete develops small channels inside the concrete increasing the porosity and making it easier for vapor to move within the matrix without inducing high internal stresses [81]. On the other hand, adding 1% steel fiber (SF) to the concrete can lead to a severe spalling problem. This effect takes place because SF elongates at higher temperatures and creates extra stresses within the concrete. Several research findings showed that reducing the percentage of SF from 1% to 0.3% or 0.5% can reduce the effect of spalling [82]. The low amount of SF does not lead to a significant effect when the SF expands. Moreover, the addition of SF to concrete has a large effect on the residual flexural stress and post-cracking behavior while cellulose or PP fibers do not have a significant influence on it. Although using 1% SF leads to a major spalling problem, it can still maintain about 15% more flexural stress at 800 °C than that of concrete with 0.3% SF because of improved concrete tensile stresses due to the increased fiber count. In addition, concrete with 1% SF can maintain its original strength at 200 °C, which could not be achieved by using 0.3% or 0.5% SF as they start losing strength directly after heating. A small SF percentage (limited fiber count) has limited contribution to the control of internal cracking. In turn, using a large percentage of steel fibers will reduce the overall strength and the modulus of elasticity of concrete [81,82]. At 500 °C, the SF fibers can be seen pulling out of the specimen, while at 800 °C, the fibers have melted completely and are not visible [81].

When PP fibers are used, the melting of the fibers at 170 °C and the slow cooling rate increase the concrete porosity and reduce the pore pressure, which lead to fewer cracks and overall similar results when compared to not using any fibers. There is no major change in the stress-strain curves of concrete with PP fibers and volumetric ratios of 1% or 2% [83]. Moreover, the maximum stress values were found in PP fiber concrete followed by SF concrete [83]. Although using SF can improve crack control, the use of PP fibers is better. This happens because the SF’s high thermal conductivity allows the heat to reach inside of the concrete more quickly, thereby reducing the temperature gradient. Increasing the percentage of SF will yield a better result in terms of residual splitting strength. This however applies only up to volumetric ratio of 1%, any percentage higher that will not lead to an increase in strength. Also, the PP fibers can lead to an increase in the residual strength only up to a certain percentage. After that, adding more PP fibers will make the concrete highly porous and reduce its strength. It was found using a hybrid fiber with volumetric ratio of 1% SF and 0.1% PP fibers is the optimum way for improving splitting strength [84]. This composition will maintain the benefits of the SF while at the same time reduce spalling due to the PP fibers.

**Figure 20:**
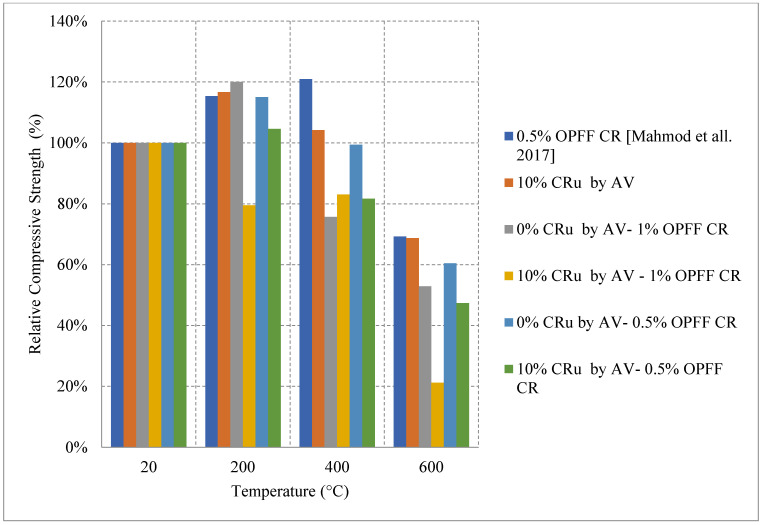
Effect of crumb rubber and palm fruit oil on the relative compressive strength of naturally cooled FRC. Cru: Crumb Rubber, OPFF: Palm Fruit Oil, CR: Cement Replacement, AV: Aggregate Volume [85].

Recycled rubber, better known as crumb rubber, is being studied as a viable option to become one of the modern constituents used in concrete that can improve thermal resistivity. Crumb rubber is used as replacement by volume fraction of fine aggregate which led to improvement of concrete behavior when exposed to elevated temperature. Concrete with 10% crumb rubber had about 20% increase in the residual compressive strength at 200 °C, as shown in Figure 20 [85]. However, it starts losing strength, reaching 70% at 600 °C. The increase in strength during the early stage of heating could be explained by the increased hydration of the cement and the enhanced bond between the rubber and other constituents within the concrete matrix. Moreover, Oil Palm Fruit Fiber (OPFF) is also a natural constituent that can be used to reduce carbon footprint and its post-fire properties are still under investigation. Unlike crumb rubber, using volumetric ratio of 0.5% of OPFF to replace cement by weight and heating at the same rate led to about 20% increase in the strength continues until 400 °C [85]. The increase might also be attributed to continuous hydration of cement; however, the resistance to early cracks within the concrete due to the addition of OPFF can also improve the compressive strength. Trying different combinations of crumb rubber and OPFF can lead to different behavior. More specifically, adding 10% crumb rubber by volume with 1% OPFF by weight of cement content leads to an 80% loss in the compressive strength at 600 °C, which is very low compared to other combinations [85]. 

**Figure 21:**
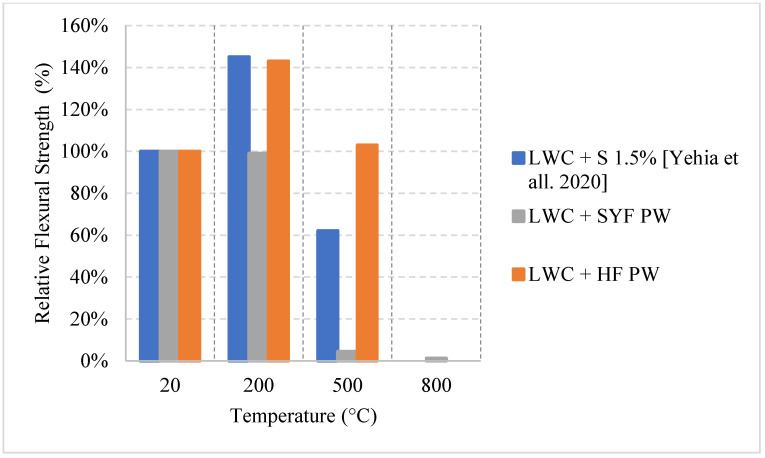
Effect of different fibers on the relative flexural strength of naturally cooled FRC. S: Steel fiber, HF: Hybrid fiber, SYF: Synthetic fiber, OR: Ongoing Research [64].

**Figure 22:**
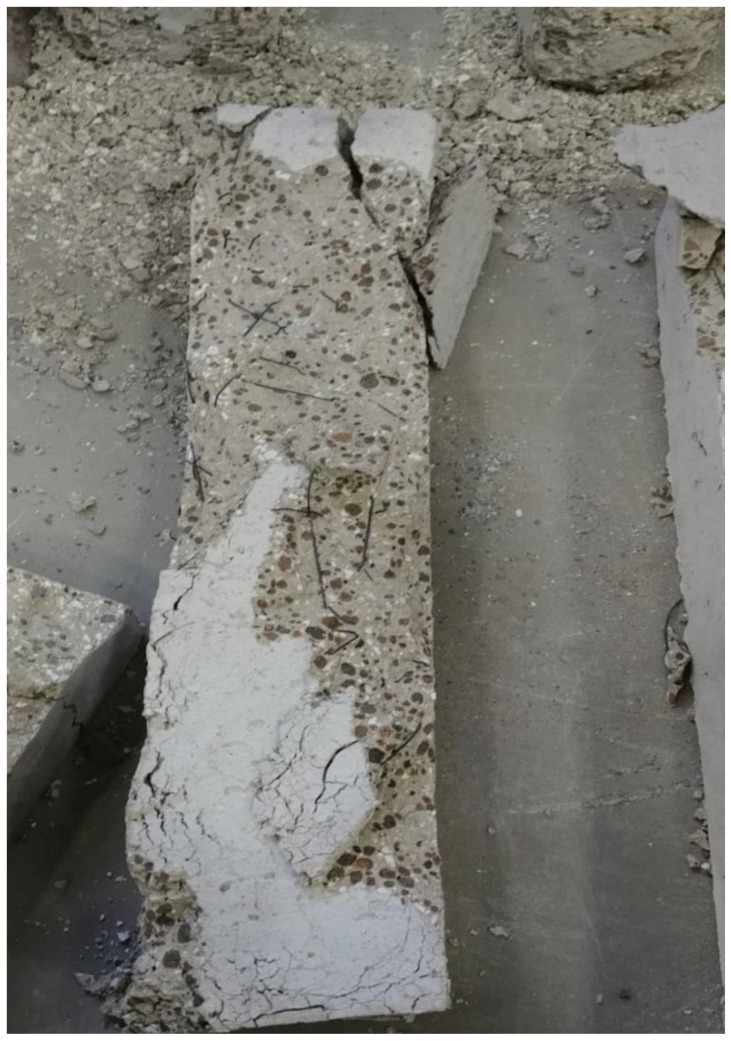
Spalled samples after temperature loading with 800 °C.

Ongoing research on fiber reinforced LWC was also done regarding the residual flexural capacity of LWC using steel and synthetic fibers. The results presented in Figure 21 [64] show that using steel or hybrid fibers yields an increase in the flexural strength at 200 °C. This increase is around 40% and is attributed to the fact that steel fibers have a higher melting point. Therefore, at this temperature, the steel fibers will still be able to control the cracks and provide the concrete with enough durability and resistance to fracture. Since the steel fibers can hold the LWC matrix together, they have proven to be better than only adding synthetic fibers. However, adding a mix of both was the best way to maintain a higher residual flexural strength. At 500 °C, the mixture containing both types of fibers were able to resist more since it is less dense than the moisture with only steel fibers. The steel fibers will increase the internal stress at that temperature due to the elongation; however, the addition of synthetic fibers was able to reduce the internal stresses and therefore the specimens were able to maintain their capacities. Figure 22 shows an example of a spalled steel fiber LWC sample. This spalling took place at 800 °C due to the severe stresses created by the steel fibers. In fact, during the heating phase, samples that had a steel fiber spalled more frequently than those without any fibers. 

#### 2.4.2. Natural Cooling inside the Furnace

**Figure 23:**
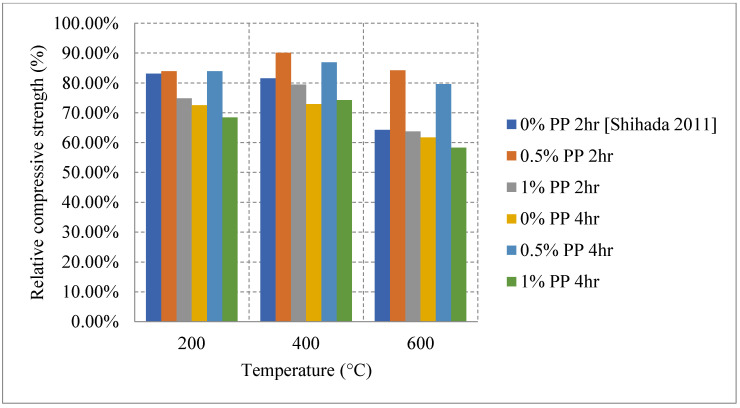
Effect of PP fibers on the relative compressive strength of naturally cooled FRC. 2 hr: 2-h exposure, 4 hr: 4-h exposure [87].

Another unique type of fibers are hemp fibers. These fibers are extracted from plants and can be integrated into the mix design to enhance the post-fire performance of concrete. Using PP or hemp fibers can increase the compressive strength by about 12%. This could be attributed to the increased bond present at lower temperatures before the disintegration of the fibers. With this type of fiber, the concrete did not benefit from the channel creation once the fibers disintegrate, since the water has already evaporated and the advantages of the channels by fiber disintegration are not significant. Nevertheless, hemp fibers were still able to improve fire resistance by reducing the propagation of cracks at 400 °C [86]. FRC, using different types of fibers, showed more losses in the modulus of elasticity, flexural strength, and the compressive strength. The literature shows that, at every temperature, 0.5% of PP fibers per volume yield better results than 0% or 1% per volume as a replacement of the total concrete mix. As shown in Figure 23 [87], both 0% and 1% of PP fibers per volume had similar results at 600 °C, and their residual compressive strengths are 64% for 2-h exposure and around 60% for 4-h exposure. The use of 1% PP fibers per volume leads to negative effects such as leaving a large empty space within the concrete once melted that can create an overall weaker structure. Therefore, its results are similar to those of 0% PP. It was concluded that 0.5% of PP fibers per volume are an optimal percentage by volume, with more than 80% of its strength retained at 600 °C after both exposure periods. To the authors’ best knowledge, there was no studies on steel fiber reinforced concrete that is cooled inside the furnace and this requires further studying.

#### 2.4.3. Water Cooling 

Although the water-cooling method is the most realistic, studies on the effects of water cooling on FRC are very limited. Water cooled concrete prepared using different percentages of PP fibers can lead to great variability in the overall behavior. It was found that water spraying the PP fiber samples for 5 min yielded different results than that quenching them in water. In fact, the longer the samples are sprayed with water, the weaker the concrete becomes. Concrete that was water sprayed for 30 or 60 min yielded the same results as water quenching. This result is different than that of naturally cooled PP fiber concrete due to the fact that water cooling leads to a thermal shock that creates additional tensile stresses that damages the integrity of the concrete matrix and leads to a weaker concrete [16]. When concrete with rubber fibers is exposed for 2-h before being cooled, the concrete tends to behave similar in the sense that there is a slight increase in the tensile splitting strength at 150 °C and an almost linear decrease after that [88]. The decrease could be attributed to the thermal shock, which induces micro cracks when water cooling is used. The increase, however, is due to the fact that at 150 °C the rubber has not melted yet and is still able to improve the residual tensile strength of concrete due to the increased bond it creates within the concrete. After 180 °C, the rubber begins to melt and its positive effects are vanished. Although a higher percentage of rubber fibers reduces the initial strength of concrete, it has the ability to retain more of its strength at very high temperatures. 

**Figure 24:**
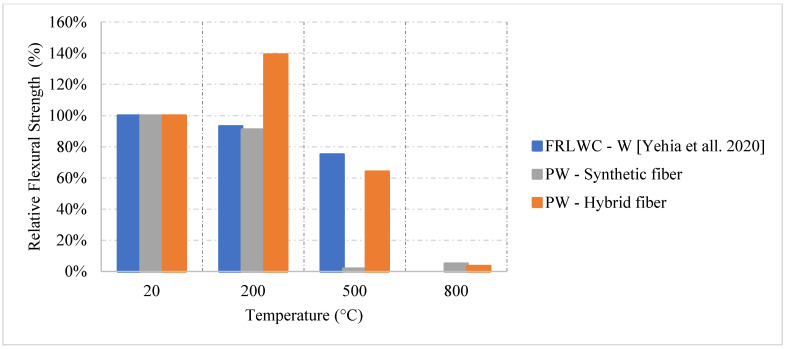
Effect of different fibers on the relative flexural strength of water cooled FRC. S: Steel fiber, HF: Hybrid fiber, SYF: Synthetic fiber, OR: Ongoing Research [64].

From the ongoing research on the subject, water cooled concrete having a combination of synthetic and steel fibers will yield the best results initially as shown in Figure 24 [64]. This is true at 200 °C because the concrete can have the benefits of both fibers. If synthetic fibers are used alone, this can increase the porosity of the matrix, which in turn reduces the concrete tensile strength. However, when both fibers are used. The steel fiber will be able to withstand more stresses at 200 °C because it had neither deteriorated completely nor elongated to a point where the internal stressed became significant. This combination yielded a strength increase even under the effect of the thermal shock. This is mainly due to the fact that the effect of both fibers created a balanced matrix in terms of stresses, allowing for the creation of additional C-S-H to add to the strength of the mix. 

The studies summarized in Table 4 [16,64,81,82,83,84,85,86,87,88] focus on steel and PP fibers. However, there is a lack of studies on FRC subjected to water cooling or cooling inside the furnace. This emphasizes the need for more research to be done around these topics. The fibers with different percentages, shapes, and sizes can change the behavior of concrete significantly if the water is cooled.

### 2.5. Ultra-High-Performance Concrete (UHPC)

UHPC is another unique type of concrete that is commonly used in bridge decks and other structures that require the concrete to have a high tensile strength. Here, it is important to note that fire in bridge decks is an entirely different scenario than fires in buildings. However, the studies in the literature use the common methods of conducting the experiment regardless. This type of concrete can yield a compressive strength between 100 and 150 MPa, which is significantly higher than all the other types of concrete. Designers could rely on this type of concrete to reduce the dimensions of the concrete elements without sacrificing durability or strength [89]. UHPC provides a high tensile strength due to its heavy reliance on steel fibers. However, its matrix is considered very dense with low porosity, low w/c ratio, and high steel fiber content, which all make it susceptible to spalling [90] compared to other types of concrete. On the other hand, it was found that the use of PP fibers increases the porosity of the concrete and in turn slightly protects against spalling [91]. There are many discussions in the literature regarding the effects of steel fibers on the spalling of UHPC specimens. Nevertheless, the overall discussion and investigation regarding the effects of changing the constituents of UHPC to improve its post-fire residual properties are still smaller that than regarding NSC or HSC. Moreover, the codes do not provide any real data regarding this type of concrete, even though it is one of the most important types when it comes to bridge deck designs. This emphasizes the need for more research to determine the extent of these effects, since UHPC is used in critical infrastructures. 

#### 2.5.1. Natural Cooling outside the Furnace

**Figure 25:**
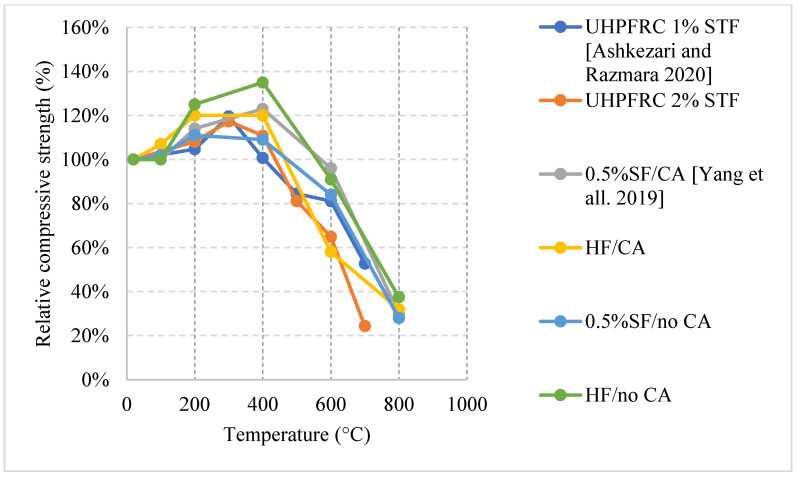
Relative compressive strength of naturally cooled UHPC with different constituents. STF: Steel Fiber, H: Heated, CA: Coarse aggregates, HF: Hybrid Fiber, CA: Coarse aggregate, SF: Silica fume, HF: Hybrid fiber, STF: Steel fiber, V: Volume [38,92].

**Figure 26:**
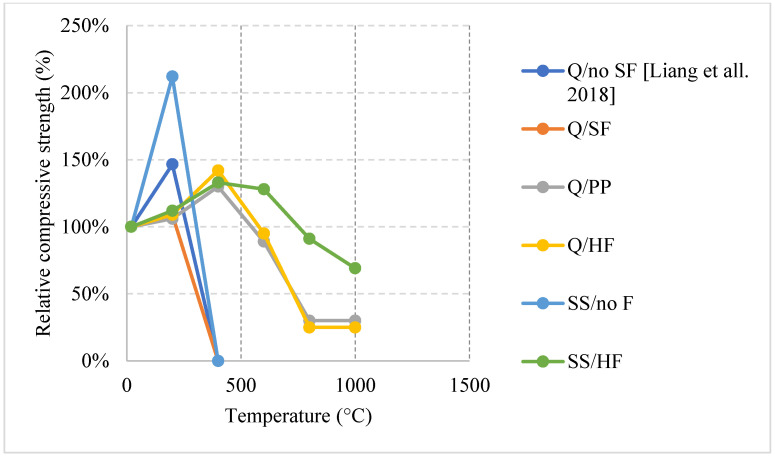
Relative compressive strength of naturally cooled UHPC (heated versus not heated). Q: Quartz, SF: Steel fiber, PP: Polypropylene fibers, HF: Hybrid Fibers, SS: Steel Slag [93].

Unlike other concrete types, the high density of its matrix can be very detrimental at high temperatures. Naturally cooled UHPC specimens tend to undergo a general trend in which the concrete breaks off or spalls; usually, concrete spalls after 700 °C [38]. However, other research efforts showed that UHPC can spall at different temperatures as well [92,93]. UHPC can lose up to 15% of its entire weight by 400 °C. This is a significant loss that takes place due to all the accumulated vapor pressure and the negative effects of the steel fiber elongation. On the other hand, UHPC can yield great properties at the early stages of heating, in fact, the increase of steel fiber as a volume fraction from 1% to 2% yielded a 12% increase in the modulus of elasticity along with the overall flexural strength [38]. Consequently, UHPC can maintain most of its strength at elevated temperatures. Moreover, as shown in Figure 25 [38,92] and Figure 26 [93], all UHPC specimens show an increase in the compressive strength between 200 °C and 400 °C. The strength increase is significant and can reach around 210% [93]. Research findings showed that using Quartz or steel slag as fine aggregates improve the performance significantly since both materials have good fire resisting properties [93]. In fact, quartz has a melting point of 1650 °C. In addition, lack of coarse aggregates reduces the effect of the expansion incompatibility between the cement paste and the coarse aggregates [93]. Nevertheless, this incompatibility increases the risk of spalling. At 200 °C, adding fibers to a high strength-fine aggregates mix reduces its residual strength properties. It can be seen in Figure 25 that using fibers with fine aggregates enhanced the performance. However, Figure 26 shows that neglecting the use of fibers and only relying on fine aggregates improved the strength at early stages. Because of quartz inversion after 573 °C, it is noted that using basalt instead of quartz improves the behavior even more [38]. However, in all cases, spalling is an issue, even when using fiber with low melting points such as PP fibers. The increase in the porosity of the UHPC does not have a significant effect on the reduction of spalling, especially when coarse aggregates are not used. This could be explained by the dense microstructure of the UHPC. When the UHPC does not spall, it still undergoes color change and cracking [38]. It was also found that the use of 2% fibers as a volume fraction increased the surface cracking [38]. The cracks were observed more clearly than when using 1% fibers. However, samples with 2% fibers showed similar weight loss to samples with 1%. From the general trend, adding more fibers will increase the surface cracking especially in UHPC given its highly dense matrix. Moreover, it was found that there is an increase in the risk of spalling for UHPC specimens with high moisture content especially when they are taken out of the curing tank, their surface wiped, and immediately tested [92]. When testing the samples after immediately taking them out of the curing tank, it was found that adding PP fibers does not reduce the risk of spalling. Moreover, there is no clear relation between the excessive vapor pressure and spalling. This area needs to be investigated thoroughly by implementing more fiber percentages with different moisture contents. 

#### 2.5.2. Natural Cooling inside the Furnace

**Figure 27:**
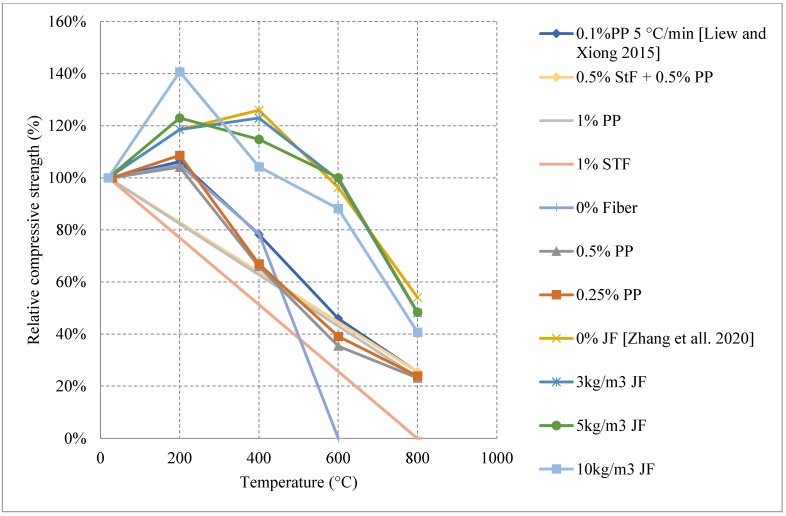
Relative compressive strength of furnace cooled UHPC with different constituents. JF: Jute Fibers, STF: Steel Fibers, PP: Polypropylene [47,94].

Concrete that is subjected to high temperatures is expected to directly lose its strength. However, most of the studies in the literature reported an increase in strength between room temperature and 500 °C. This increase is mainly attributed to the migration of excess water in the pores, which creates extra hydrates that improves the bond characteristics and creates shrinkage. It is also stated that cement rehydrates at 200 °C and creates additional gel that can enhance the bond as well. The increase of the compressive strength varied among the studies found in the literature, as shown in Figure 27 [47,94]; for instance, all UHPC samples that used jute fibers witnessed a strength increase before 500 °C. However, 10 kg/m^3^ of natural jute fibers was the most efficient, as it can lead to a 40% increase at 200 °C. When jute fibers shrink, they leave behind pores but are still able to perform their intended use and improve the overall strength of concrete [94]. In addition, this amount of jute fibers also proved to prevent spalling of UHPC by shrinking at the early stage of heating, creating many pores in the UHPC matrix, and increasing the overall porosity of the concrete. On the other hand, the use of PP can allow for a strength increase, but it is not as significant as that created by jute fibers [44]. This could be because at early stages the PP fibers are expanding and increasing the internal pressure inside the concrete matrix. They would still perform relatively well relatively; however, they would only allow for a small 10% increase in strength. In fact, Jute fibers burn completely at 107 °C while PP fibers are just expanding and did not even start to melt. 

**Figure 28:**
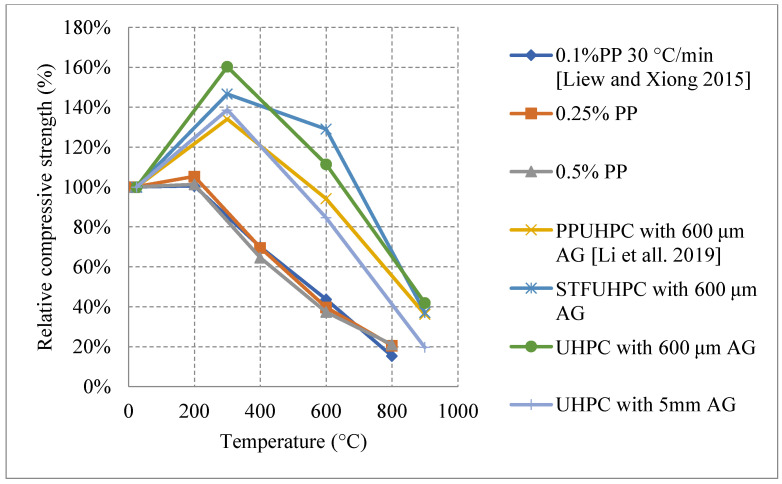
Relative compressive strength of furnace cooled UHPC with different constituents 2 [47,95].

Figure 28 [47,95] represents other UHPC samples that yield an increase in strength. Although the increase is also said to be due to water/binder phenomena, there are some other reasons related the use of fibers [47,95]. It was found that neglecting the use of fibers and entirely relying on very fine aggregates (600 μm) proved to be the best in terms of residual strength increase during the early stage of heating. The strength increases; however, they are not far from each other. Nonetheless, removing fibers could have reduced the development of eternal stresses while maintaining the compressive strength of UHPC. The downside to removing fibers is that the UHPC would fail completely in tension. UHPC was better off when using steel fibers than jute fibers or PP fibers. The use of PP fibers and larger aggregates increases the permeability of concrete while steel fibers do not [96]. This complicates the results because it changes the perspective of the benefits of increased porosity. In many cases, the increased porosity yielded better results. However, in the case of steel fibers, the UHPC seems to maintain its dense structure while at the same time its strength. Although spalling is an undeniable issue in the use of steel fibers, their nature allows them to control crack propagation and reduce the negative effects of concrete deterioration at early temperatures [44].

#### 2.5.3. No Cooling

**Figure 29:**
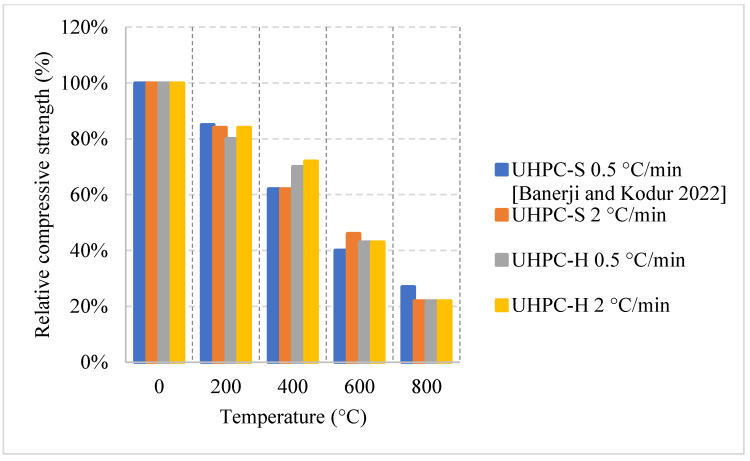
Relative compressive strength of UHPC under different heating rates. S: Steel Fiber, H: Hybrid between steel and Polypropylene Fibers [97].

**Figure 30:**
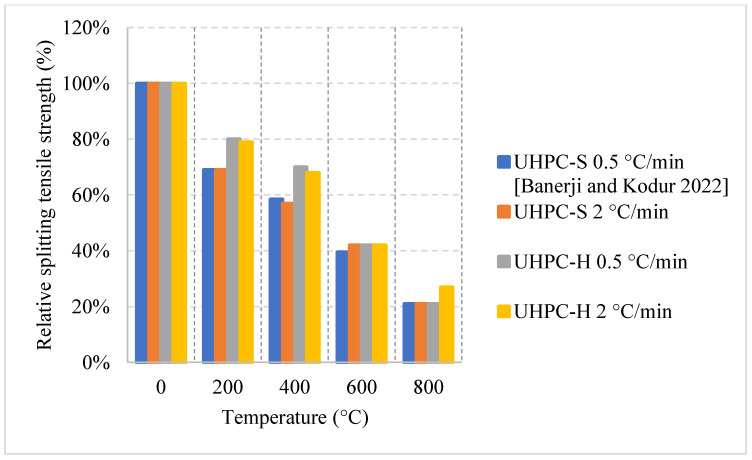
Relative splitting tensile strength of UHPC under different heating rates. S: Steel Fiber, H: Hybrid between steel and Polypropylene Fibers [97].

For this regime, the UHPC samples have been heated to different temperatures under two different heating rates. Figure 29 and Figure 30 [97] show the effect of steel fibers and hybrid fibers (steel and PP fibers) and the effect of different heating rates on the relative compressive and splitting tensile strengths. The study states that the effect of PP fibers on UHPC is negligible. The comparison was mainly between steel and hybrid fibers. Both the compressive and splitting tensile strengths start to decrease as soon as the temperature is increased. The difference in both cases at 400 °C was attributed to the fact that the PP fibers in the hybrid mix deteriorate at around 170 °C [97]. This improves the permeability of the concrete and reduces the deterioration of the microstructure. The constant drop in strength is dominant in UHPC, since at higher temperatures, the highly dense matrix is unable to withstand the excessive built-up pressures.

Table 5 [38,47,92,93,94,95,97] shows some of the important parameters for each study. Some studies use coarse aggregates while others do not. Since the studies use almost the same constituents, the aggregates are the main difference that can lead to a significant change in the behavior of UHPC.

### 2.6. Self-Consolidating Concrete (SCC)

SCC is a relatively new concrete in the world of civil engineering. Since SCC is highly workable and does not need vibration, civil engineers use SCC to reduce the construction time and make it easier for placing and finishing of congested formwork. However, SCC does increase the cost of construction and requires more quality control. In addition, SCC is made from a wide range of mixes, and there is no specific mix that represents it [98]. Therefore, the use of this type of concrete is still limited compared to other types of concrete. In terms of fire, SCC is able to yield similar results to NSC [99]. Similar to other types of concrete, it is shown in the literature that the overall behavior of SCC changes with a change in its constituents. For instance, changing the fillers from limestone to Quartz or Basalt will change the mechanical properties of SCC under fire. Combinations of different fillers can also change the behavior of SCC [100]. A more in-depth explanation of the results found in the literature are shown in the following section. 

#### Natural Cooling outside of the Furnace

*Normal weight SCC (NWSCC):*

Natural cooling outside the furnace is the common cooling method used by most of the studies found in the literature. Moreover, there is a variety of SCC types that are studied. These types cannot be compared directly because each of them has an entirely different composition and different proportions of materials. Therefore, mixtures within the same category were included in the discussion. 

**Figure 31:**
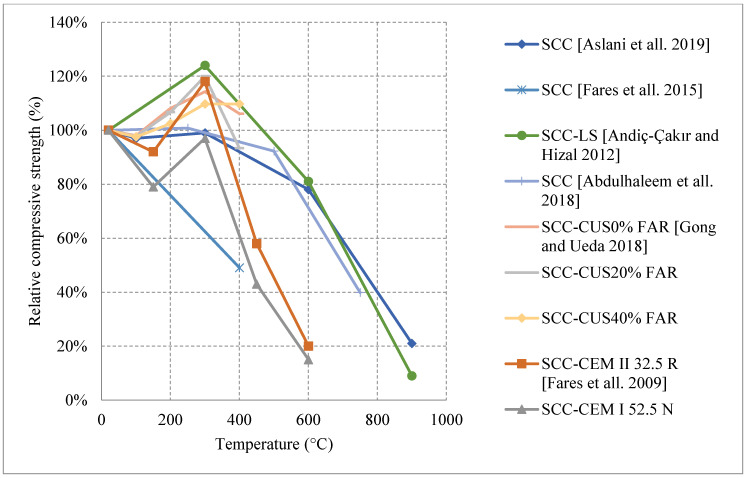
Relative compressive strength of naturally cooled SCC with different constituents. LS: Limestone, CUS: Copper Slag, FAR: Fine Aggregate Replacement [75,101,102,103,104,105].

**Figure 32:**
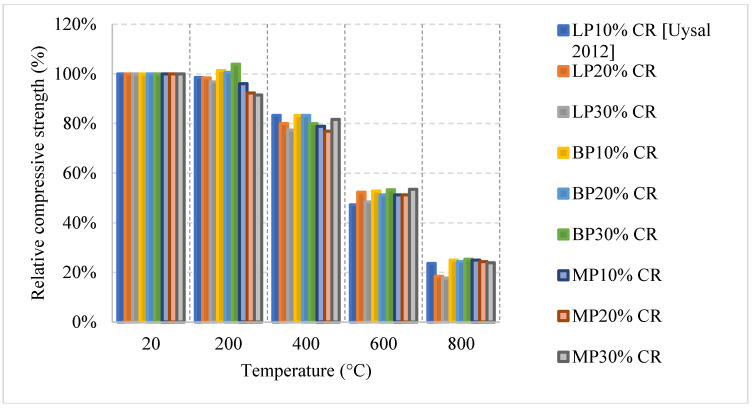
Relative compressive strength of furnace cooled SCC with different constituents 2. LP: Limestone powder, BP: Basalt powder, MP: Marble powder, CR: Cement Replacement [106].

Behavior of NWSCC is represented in Figure 31 [75,101,102,103,104,105]; the wide variety of the results is a consequence of the difference in the constituents used in the making of the SCC samples. NWSCC seems to show an increase in residual compressive strength mostly between 200 °C and 400 °C. In fact, this increase could be up to 24% at 400 °C [102]. In some cases, the strength increase could be due to the loss of interlayer water and water from calcium sulphoaluminate cement and sulpho-aluminate hydrates at these relatively moderate temperatures, or a result of the rehydration due to continuous curing similar to steam curing. The rehydration can add up to 20% more strength at 300 °C and is very efficient since SCC contains a higher amount of binders than other types of concrete. There are scenarios in which the SCC losses around 6.5% of its mass between 150 °C and 300 °C and still show an increase in strength [105]. Another reason leading to the strength increase is assumed to be the creation of shorter and stronger Siloxane elements (Si–O–Si) that have bigger surface energies. This creation is a result of the loss of bond between silanol groups and water when subjected to high temperatures [105]. Some mixes proved that the use of cooper slag (CUS) as a fine aggregate would yield an increase in the porosity and a lower modulus of elasticity. The residual modulus of elasticity of SCC containing 20% and 40% copper slag drops quickly between 300 °C and 400 °C due to the micro cracks that form around the fine aggregates [104]. In addition, when using 20% CUS, there was a sharp drop in the residual compressive strength after 300 °C due to the presence of these cracks. However, adding 40% CUS was found to be the most optimum as it decreases the rate of strength loss in most of the SCC and makes it the most durable compared to other percentages [104]. This is mainly due to its low water absorption. In general, CUS proved to reduce the natural compressive strength of SCC, but it was excellent in terms of residual properties. The lower strength is attributed to the weak bond between the CUS aggregates, and the cement paste and the fact that there is bleeding water around the aggregate surface. In the worst cases, the SCC gradually became weaker at the beginning of the heating process, and after 350 °C the rehydration of lime was accompanied by an expansion that decreased the compressive strength and the overall behavior significantly. Moreover, Figure 32 [106] shows that the use of limestone powder, basalt powder, or marble powder in different percentages will yield almost the same behavior after 400 °C. However, there is a notable difference between using 30% Basalt powder and the others. This could be attributed to the variety of constituents whose behavior can impact the behavior of SCC in different ways. Studies on SCC are still limited and the effects of constituents on this type of concrete needs to be studied thoroughly.

*Lightweight SCC (LWSCC):*

A quick comparison between LWSCC and NWSCC would show that NWSCC performs better before 600 °C. This is because lightweight aggregates absorb water at a faster rate. During the heating process the water evaporates and the bonds between aggregates is significantly damaged. However, after 600 °C the NWSCC matrix starts to deteriorate and develop many internal cracks that weaken the compressive strength of the concrete. Nonetheless, in terms of spalling, using lightweight aggregates yields better results since they add much less internal pressures at higher temperatures than normal weight aggregates. 

**Figure 33:**
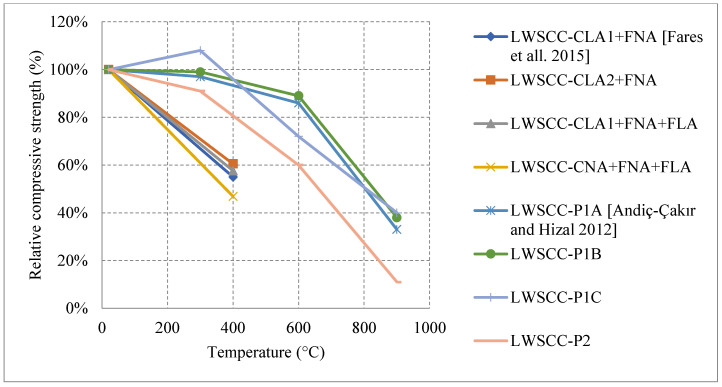
Relative compressive strength of naturally cooled LWSCC with different constituents. CLA = coarse lightweight aggregate; CLA2 has smaller average particle size than CLA1; CNA = coarse normal-weight aggregate; FLA = fine lightweight aggregate; FNA = fine normal-weight aggregate; P1A = mixture having a 0.42 w/p ratio; P1B; mixture having a 0.38 w/p ratio; P1C = mixture having a 0.35 w/p ratio; P2 = mixture having a 0.34 w/p and different proportions of materials [101,102].

**Figure 34:**
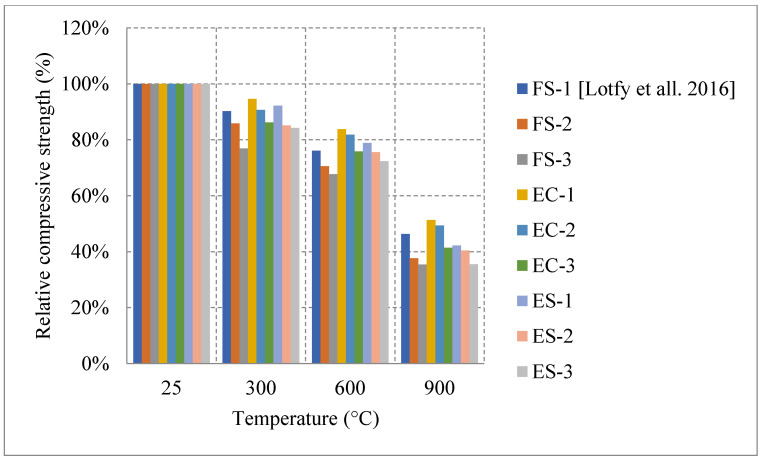
Relative compressive strength of naturally cooled LWSCC with different constituents 2. 1 = Casting by a pump injection system; 2 = Suitable for many normal applications; 3 = Suitable for vertical applications in very congested structures, structures with complex shapes, or for filling under formwork; FS = furnace slag; EC = expanded clay; ES = expanded shale [107].

Constituents used in making LWSCC change its behavior in several ways, as shown in Figure 33 [101,102]. Some LWSCC mixtures showed a direct drop in compressive strength and their performance was relatively weak. The linear drop in strength was caused by the loss of bound water between cement hydrates and a chemical decomposition that led to a significant change in the microstructure, creating many internal micro cracks [101]. These micro cracks were more prominent in LWSCC that uses a mixture of both lightweight and normal weight aggregates. This is because adding normal weight coarse aggregates led to a weaker bond at higher temperatures and increased the porosity much more than just relying on a mixture of lightweight coarse and fine aggregates. Moreover, using a large amount of coarse aggregates is sometimes better depending on the mix [101]. The difference in the post-fire results could also be due to the different thermal conductivities of different aggregate types. Moreover, there were LWSCC samples that performed much worse even when the coarse aggregate content was little [101]. This was because other constituents played a big role in its loss of strength. For instance, it was clear that air content plays a major role in reducing the compressive strength. Consequently, adding more AEA and increasing the air percentage can negatively affect the integrity of the matrix making it very fragile and susceptible to damage at higher temperatures. LWSCC sample with a small amount of AEA and a small water/powder (w/p) ratio performed better at the early stages of heating [102]. This is because having such factors would reduce the permeability and create the better bonds between constituents. However, at much higher temperatures, the best mixes were those with higher w/p ratios and small air percentage. Having a porous LWSCC matrix would allow it to be better at resisting the thermal effects at high temperatures. For example, Figure 34 [107] shows that, when the effects of some aggregates at 300 °C on the behavior of SCC are directly compared, it was found that using expanded clay (EC) is better than using expanded shale (ES) or furnace slag (FS) [107]. This could be attributed to the hardening of the hydrated cement paste and the movement of cement gel layers closer together when water evaporates. In terms of mass loss, SCC using EC or ES lost more mass than SCC with FS. This is mainly due to their high absorption and high porosity features. However, the denser matrix created by the addition of Furnace slag (FS) aggregates led to higher internal pressures as well as many micro cracks at very high temperatures. In general, studies that highlight the effect of constituents on the thermal properties of LWSCC are scarce and there is a need to study the extent of these effects to avoid any failure that could happen in case of a fire.

*Fiber reinforced SCC (FRSCC)*

**Figure 35:**
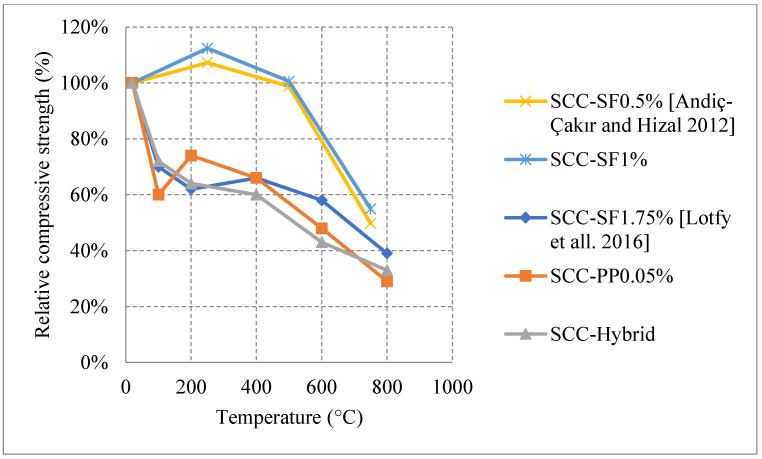
Relative compressive strength of naturally cooled FRSCC with different constituents. SF: Steel Fiber, PP: Polypropylene Fibers [102,107].

**Figure 36:**
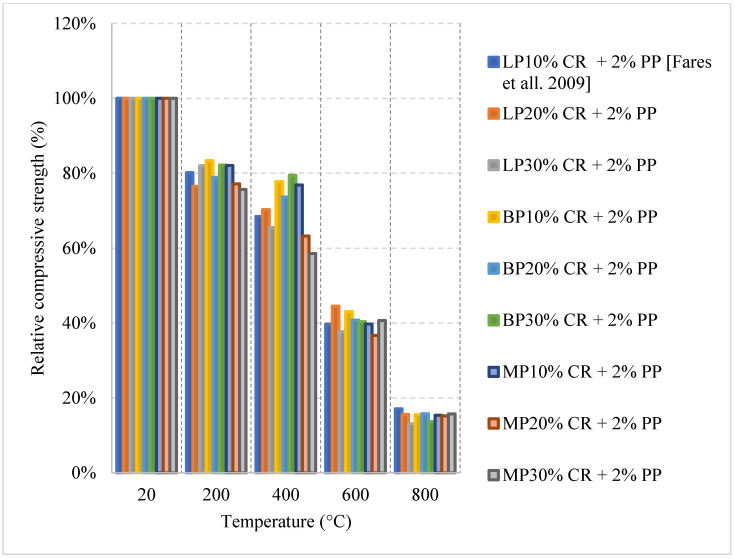
Relative compressive strength of Furnace cooled FRSCC with different constituents. LP: Limestone Powder, BP: Basalt Powder, MP: Marble Powder, PP: Polypropylene Fibers, CR: Cement Replacement [105].

The addition of fibers to the SCC matrix yielded distinct results, as shown in Figure 35 [102,107] and Figure 36 [105]. The FRSCC is thought to improve the behavior of concrete subjected to elevated temperatures. On one hand, using 0.5% or 1% of steel fiber (SF) per volume of concrete causes an increase in compressive strength reaching 10% to 15% at 300 °C [103]. Furthermore, this addition can leave the SCC with around 50% of its original strength at 800 °C. The increase in strength is attributed to the rehydration of cement as water migrates from the concrete, and in addition to the slight expansion of SF at lower temperatures, this rehydration can provide the concrete with a more compact and tighter matrix. This can slow down the formation of cracks improving the bond between the fiber and surrounding concrete. The addition of SF to SCC in such a case proved to be even better than its normal counterpart without SF [103]. However, the drop-in strength happens when SF starts to totally melt, increasing the porosity of the concrete to high levels in which its durability drops drastically. On the other hand, using 1.75% of SF, 0.5% of PP fibers per volume of concrete, or a hybrid of both led to a very steep drop in strength followed by a gradual decrease [108]. In the case of PP fibers, this fast decrease is a result of melting of these fibers at low temperatures. The melting leads to a rapid mass loss and creates many micro channels that get filled with vapor, which in turn reduces the internal pressures unless the melting blocks the vapor. In all cases, the initial loss of strength is also a result of the moisture loss as well as the disintegration of C-S-H. However, the change of fiber content made no difference in some cases and the behavior of concrete was totally attributed to other constituents such as the aggregates or the cement used [103,108]. In addition, it was found that using Basalt powder can yield better residual results than adding limestone or marble powders. This agrees with the concrete mixes that did not have any fibers added to them. Nonetheless, there is a direct drop in strength due to the internal stresses created by the addition of PP fibers. At 400 °C, a part of the bond between silanols groups and water is lost. This in turn leads to the creation of shorter and stronger siloxane elements (Si–O–Si) with larger surface energies. This can lead to the strength increase in concrete [106]. The lack of studies on FRSCC highlights the need for more research to be done on the effects of fibers on SCC. 

Table 6 [75,101,102,103,104,105,106,107,108] summarizes several studies found in the literature. The summary included constituents used in each study alongside other parameters. In most of the studies, the heating rate was at 5 °C/min, but the main differences are the w/c ratio and the constituents used. Evidently, there is a wide variety of effects within each mixture that was used in these studies. However, studies on SCC behavior at high temperatures are very scarce compared to other more common types of concrete. 

### 2.7. Geopolymer Concrete (GPC)

Geopolymer concrete is a modern type of cementitious material that relies on the use of industrial by-products such as slag, rice husk ash, silica fume, or fly ash. It has excellent resistance to fire, durability, and thermal properties due to its stable chemical structure. The gels produced through the polymerization and pozzolanic reactions control the quality of the geopolymer composite. Geopolymer concrete is able to yield higher stresses on the stress strain diagrams when compared to NSC [48,109]. This was mainly attributed to the geo-polymerization of the matrix. But it could, to a limited extent, be because of the expulsion of moisture from the geopolymer gel [109]. With the continuous reliance on NSC and other types of concrete, the use of cement is proving to be detrimental to the environment. This is due to the high carbon quantities released into the atmosphere during the production of cement. In fact, cement contributes to 7% of the overall carbon emissions [110]. With the growing issue of climate change, it is clear from the literature that there is a growing effort made by engineers to reduce the use of cement and rely more on by-product materials such as fly ash. Since GPC totally replaces cement with other material additions to certain solutions, it is a more sustainable form of concrete. In addition, in an effort to improve the sustainability of concrete, the use of recycled aggregates instead of normal ones was studied. It was found that recycled aggregates made the concrete more permeable, which will improve resistance against spalling. However, it significantly reduces the compressive strength of concrete [111]. In terms of spalling, GPC tends to be very efficient at resisting its effects. GPC could also be exposed to elevated temperatures for two hours and show minor signs of spalling. This makes it much better than NSC and other types of concrete [112]. Moreover, cracking is also less in GPC than in NSC. It was found GPC develops hairline cracks after 600 °C while NSC does so at 200 °C [113]. GPC can produce a similar compressive strength to NSC, fair workability, low shrinkage, improved corrosion resistance, and most importantly low thermal conductivity. Therefore, it is important to study its post-fire properties and know what are the constituents that will help it maintain its qualities but also be able to resist the effects of fire. Other research efforts focused on introducing novel materials such as aerogel, hollow glass microspheres (HGMs), or expanded polystyrene [114,115,116] to enhance the concrete properties when exposed to elevated temperature. The HGMs have low specific heat, which increases the thermal insulation property of the concrete [115]. Moreover, the aerogel has proven to yield a higher residual compressive strength at 800 °C. At that temperature, the concrete with 50% and 75% aerogel being used as aggregate replacement per weight have an 80% and 66% residual compressive strength, respectively. On the other hand, the samples with 0% and 25% replacement by weight have a 20% and 45% residual compressive strength; respectively [116]. This is because using aerogel increases the porosity of the concrete which in turn reduces the internal stresses that are induced during the heating process.

#### 2.7.1. Natural Cooling outside the Furnace

**Figure 37:**
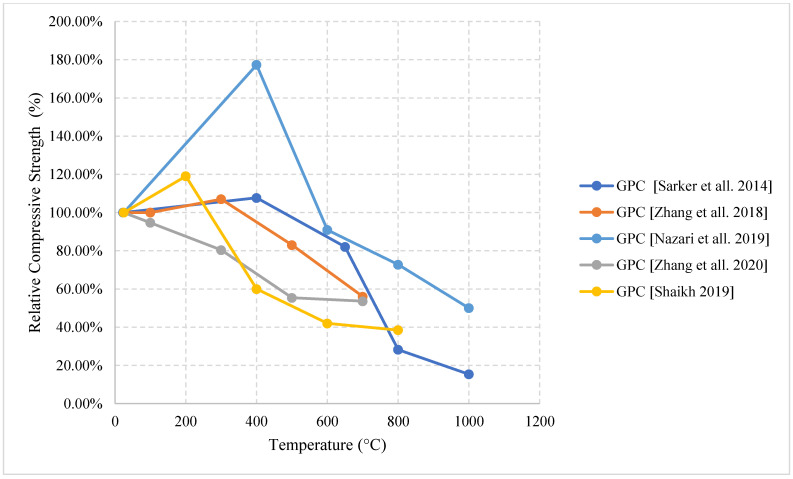
Relative compressive strength of naturally cooled GPC with different constituents [39,43,44,117,118].

**Figure 38:**
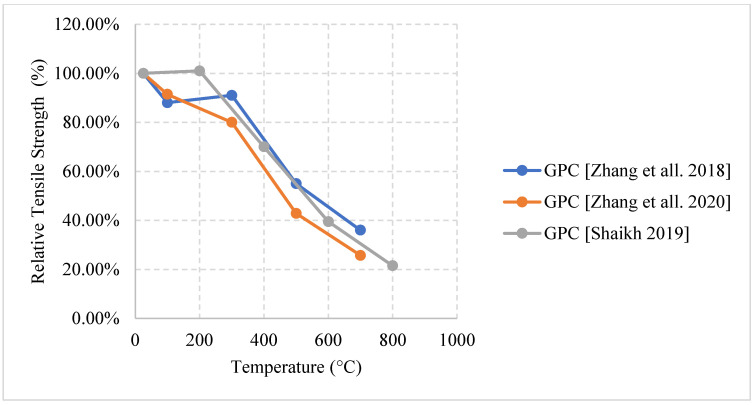
Relative tensile strength of naturally cooled GPC with different constituents [43,117,118].

When naturally cooled, GPC is likely to show a strength increase in the compressive strength between 200 °C and 400 °C as shown in Figure 37 [39,43,44,117,118]. This is true because of the sintering reaction of unreacted fly ash that can participate geo-polymerization. This means that the GPC will develop tighter bonds between its constituents which will eventually crate a stronger matrix that is able to resist higher temperatures. As shown in Figure 38 [43,117,118], the GPC does not show any significant increases in the tensile strength and starts to lose strength almost instantly due to a higher degradation under the splitting tensile loading condition. While mass loss in GPC is similar to NSC, spalling is significantly less in GPC. Very rarely did naturally cooled specimens undergo spalling as found in several studies in the literature [36,43,111]. Only at extremely high temperatures such as 800 °C or 1000 °C, vapor pressure can increase to high levels that can lead to spalling of GPC samples. However, GPC has a higher sorptivity coefficient that indicates a more porous structure; the increase in sorptivity is due to the chemical decomposition of the binders. An enhanced pore structure and a better micro-crack development can release some of the vapor pressure, reduce thermal stresses, and reduce the risk of spalling. 

#### 2.7.2. Water Cooling

**Figure 39:**
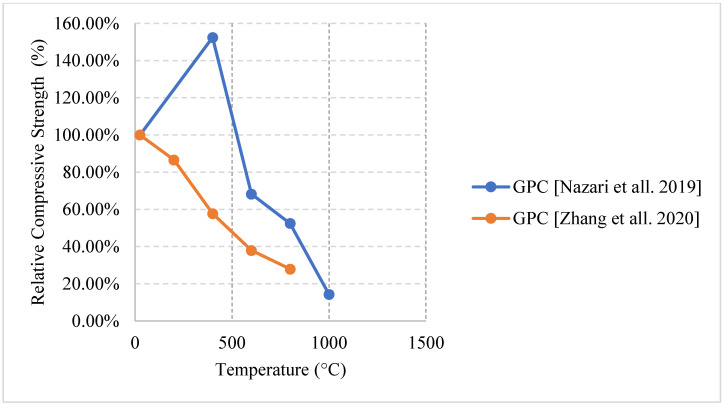
Relative compressive strength of water-cooled GPC with different constituents [44,117].

Water cooled GPC specimens yield lower results than those naturally cooled ones. However, there is an abnormal increase in strength behavior, as shown in Figure 39 [44,117]. This behavior was also present in the naturally cooled section; however, the increase is only up to around 50% rather than around 80% [39,44]. This behavior is attributed to the increased use of alkali activators, such as sodium silicate and sodium hydroxide solutions, that allow for an enhanced compressive strength. This, however, leads to a reduction in the internal pores that can increase the risk of cracking and spalling [44]. Another study that used both of these solutions did not yield an increase in strength when quenched in water [118]. This raises more questions regarding the percentage of alkaline activators used in the GPC mixture. Just like every other type of concrete, GPC cracks more when water cooled due to a sudden change in the stress difference between the surface and the internal portion of the concrete. Cracking can also take place when fly ash is synthesized with sodium alkali activators. This makes the fly ash contract and create hairline cracks [118]. 

Table 7 [39,43,44,117,118] shows that the mixes within the literature vary mostly by the additions used in the mixtures. The fine and coarse aggregates still play a role. However, it is not as significant as the role played by the additional binders and alkali activators. 28-day curing is also predominantly used, since it is the most along with a heating rate of 5 °C/min. It is also clear that there is a lack of studies surrounding this type of concrete especially when exposed to elevated temperatures.

### 2.8. Elastic Modulus 

**Figure 40:**
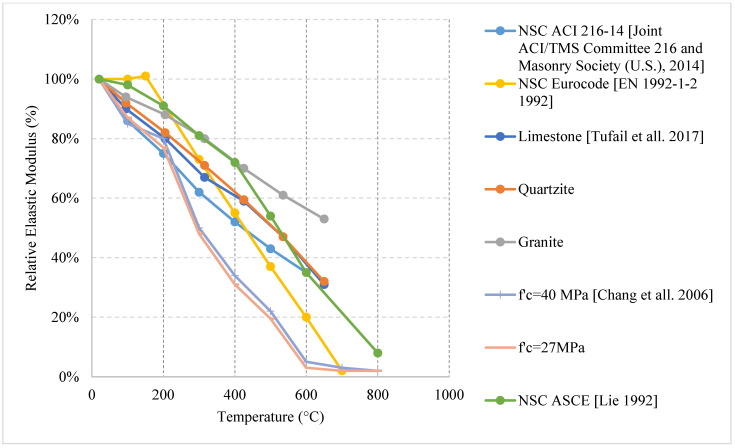
Relative Elastic modulus of different naturally cooled NSC mixes [4,5,46,48,119].

**Figure 41:**
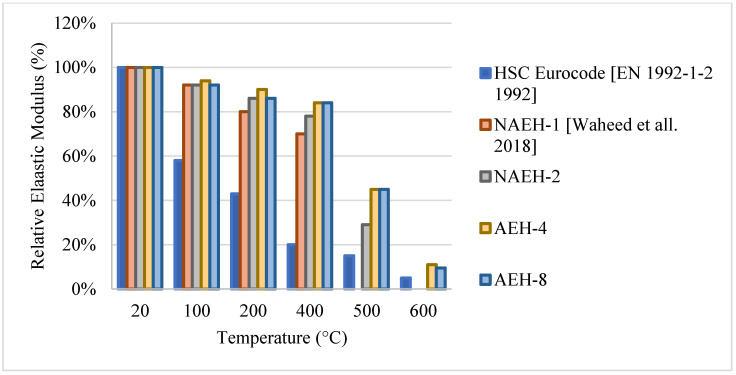
Relative Elastic modulus of different naturally cooled HSC mixes [76].

**Figure 42:**
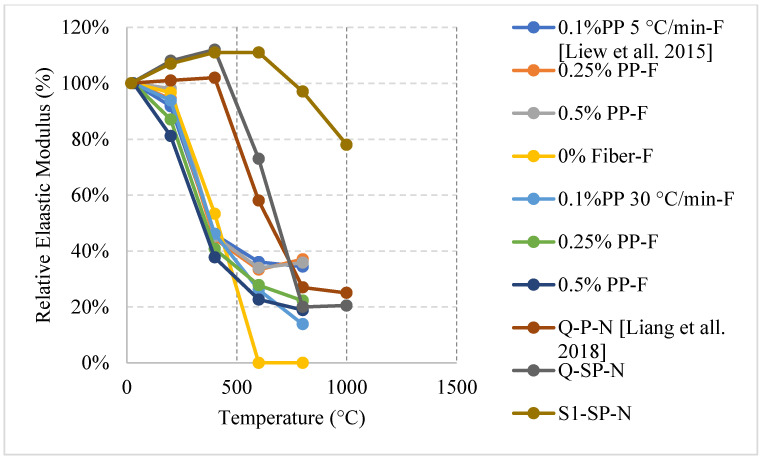
Relative Elastic modulus of different naturally/furnace cooled UHPC mixes. N = Natural cooling, F = Furnace cooling, S = steel fibers, P = PP fibers SP = Steel and PP fibers, Q = Quartz sand, S1 = Steel slag [47,93].

**Figure 43:**
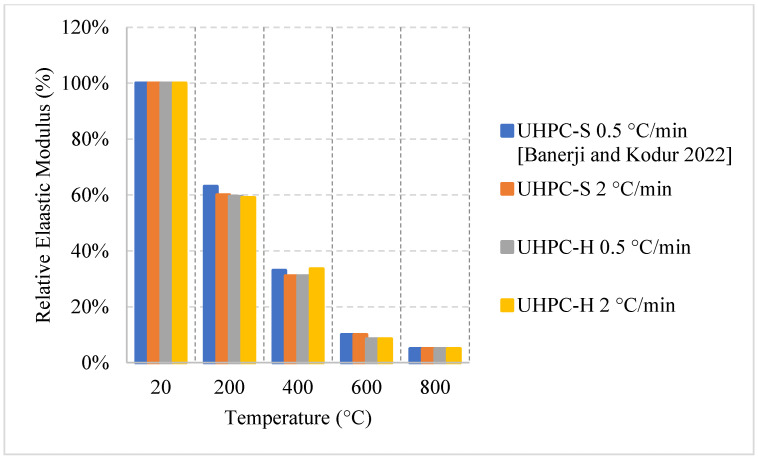
Relative Elastic modulus of different UHPC mixes without cooling. S = Steel fibers, H = hybrid [97].

**Figure 44:**
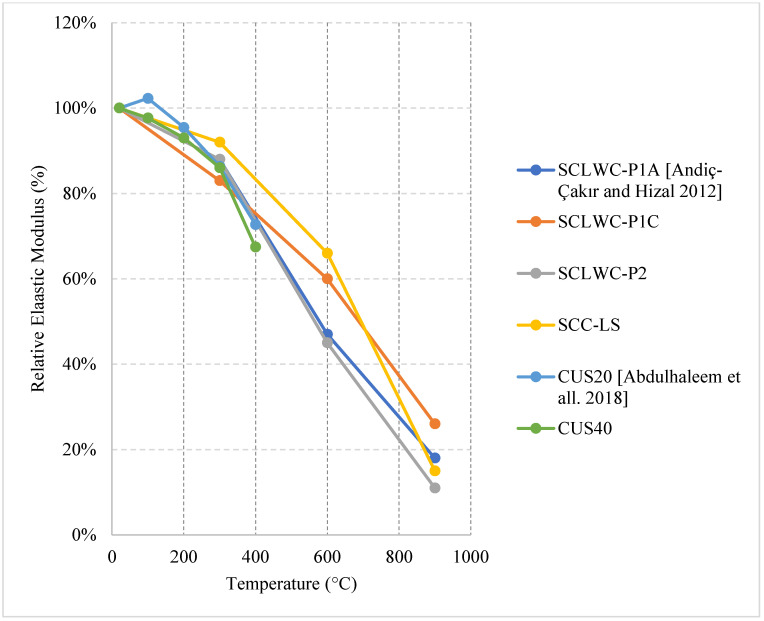
Relative Elastic modulus of different naturally cooled SCC mixes. P1A = Pumice aggregates (4–16 mm) with 0.42 w/c ratio, P1C = Pumice aggregates (4–16 mm) with 0.35 w/c ratio, P2 = Pumice aggregates (4–8 mm and 8–16 mm) with 0.35 w/c ratio, LS = Limestone aggregates, CUS20 = 20% copper slag replacement, CUS40 = 40% copper slag replacement [102,103].

**Figure 45:**
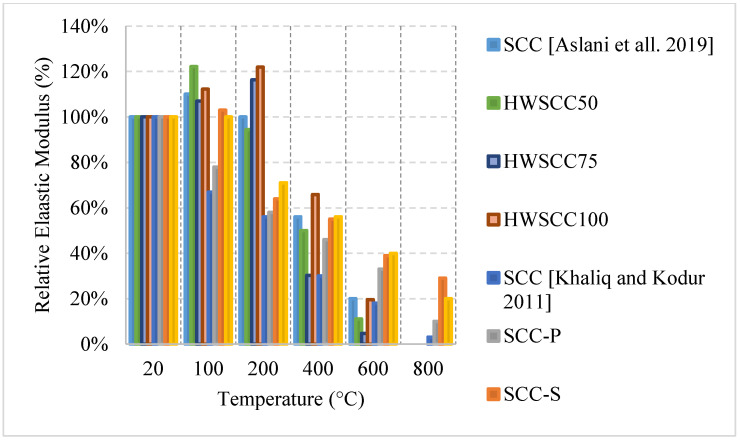
Relative Elastic modulus of different naturally cooled SCC mixes. S = Steel fibers, P = PP fibers, H = Hybrid, 50, 75, and 100 = Heavyweight magnetite aggregate with 50%, 75%, and 100% replacement ratio [75,108].

The elastic modulus of concrete varies depending on the type of concrete as well as several other factors. From the literature, data regarding each type of concrete is relatively scarce when compared to the data on residual compressive or tensile strength. It is also clear that most of the studies discussing the residual elastic modulus do so when naturally cooling their specimens. This is highlighted in the following figures. For NSC concrete that is naturally cooled, the data presented in Figure 40 [4,5,46,48,119] is close to the EN 1992-1-2 Eurocode, ACI 216-14, and the ASCE [119]. Although, the granitic mixture showed a significantly higher residual elastic modulus at high temperatures. This is because the granite aggregates have the highest resistance towards abrasion and can yield the lowest increase in the intracrystalline crack length and crack width. Moreover, Figure 41 [76] shows a large difference between the HSC results presented by the Eurocode and another study on air-entrained and non-air-entrained HSC. The results of NSC and HSC show that the design equation in Eurocode is not suitable to predict the strength of these concretes under elevated temperatures. The largest amount of data on the residual elastic modulus comes from UHPC and SCC. As shown in Figure 42 [47,93] and Figure 43 [97], the data is scattered in such a way that it is close within the same study. Yet when compared to different studies discussing the same type of concrete, there is a large difference. For instance, in Figure 43 on natural/furnace cooled UHPC, the elastic modulus is always larger at every temperature when naturally cooled. Moreover, concrete with steel slag mixed with steel and PP fibers yielded the highest residual modulus at 80%. This could be explained by the higher thermal stability of the steel slag when produced under 1650 °C and a similar chemical composition to that of cement, making it improve the compatibility between the cement and the fine aggregates [93]. This allows it to have a higher compressive strength and a higher elastic modulus. With regard to SCC, changing the size of pumice aggregates added to SCLWC can give similar results to SCC with limestone or copper slag during the early stage of heating. However, the difference increases at 600 °C and the limestone aggregates are able to keep around 70% of the original elastic modulus as shown in Figure 44 [102,103]. This is much higher the other mixes using lightweight aggregates because of the higher original compressive strength that the limestone provides. In Figure 45 [75,108], there is an increase in the elastic modulus when adding heavyweight aggregates. This was attributed to the rapid drying that is accompanied by the additional hydration of cement creating more C-S-H gel. After 400 °C, the decrease becomes clear since the replacement of natural aggregates with heavyweight magnetite aggregates can reduce the ability of cement to resist deformation, owing to the highly crystalline microstructure of the heavyweight aggregates containing weak planes [93]. It is important to note that the codes [4,5] do not provide any real equations or data regarding UHPC, GPC, SCC, or any unique type of concrete. They are only representative of NSC and HSC given certain types of aggregates only; detailed discussion is provided in the numerical analysis section. 

## 3. Numerical Analysis from the Literature

When it comes to the discussion of the effects of fire on concrete, there are many technical reports, codes, and standards that provide guidance for the designers. Several documents regarding this topic are presented in Table 8 [4,5,119,120,121,122,123,124,125,126,127,128,129,130]. The design of civil engineering structures is split into three categories within these codes. The three categories are fire testing, prescriptive methods, and performance-based methods. The fire testing is based on exposing the concrete specimen to service condition loadings under certain supports that would replicate a real-life scenario. 

The heating of such samples mainly follows certain heating curves provided by different standards [131]. Curves such as the hydrocarbon (HC) and hydrocarbon modified (HCM) curves are used to represent the combustion of hazardous materials. Rijkswaterstaat (RWS) and RABT/ZTV curves are intended to be used for the design of tunnels only [132]. The ISO 834 and the ASTM E119 curves are the most followed curves in the literature since they represent the heating scenario that takes place on a regular building structure. Next, the prescriptive method is simpler and it discusses the cover to reinforcement criteria for protection against fires. Due to its simplicity, it the most used method by the designers. Last, the performance-based method requires the designers to develop finite element analysis to check whether the design satisfies the fire design criteria [133,134]. 

Although there are several codes that provide guidance on the design of concrete against fire, the ACI 216-14 [4] and the Eurocode codes [5] are the most developed. Those two codes provide data on the residual mechanical properties of concrete and some graphs that represent the effects of fire based on the residual, stressed, and unstressed regimes. For instance, figures provided by the ACI 216-14 [4] show the difference in the compressive strengths between the three regimes for siliceous, carbonate, and semi-lightweight aggregates. Clearly there is a difference in the three regimes. The Eurocode [5] also provides tables regarding the relative mechanical properties of concrete using siliceous or calcareous aggregates. These codes also provide guidance on the cover to reinforcement requirements. However, these codes are not representative of the large variety of mixes that today’s designers are implementing. For instance, they do not cover the effect of using different cementitious materials or fibers. This is a major challenge since many designers add GGBS, silica fume, fly ash, and steel, PP, or synthetic fibers to the concrete. Moreover, they do not cover the effect of different heating scenarios. In fact, the Eurocode [5] indicates clearly that “*Possible strength gain of concrete in the cooling phase should not be taken into account*” [5]. This goes against what was presented in this study, since many concrete types and mixes can yield a very large strength gain between 20 °C and 300 °C, and they should be taken advantage of rather than neglected. In addition to that, the Eurocode [5] does not differentiate between stressed and unstressed regimes. It is important to note that these codes use a small pool of data. The ACI 216-14 code [4] lacks comprehensiveness, and the Eurocode’s equations and graphs are based on data that was extracted from studies that use materials only native to Europe. In addition, the Australian standard AS 3600:2018 [120] sheds light on the topic of fire and gives insight regarding the requirements of fire resistance. It discusses fire resistance periods and tabulates them in relation to the adequacy of simply supported beams. The Australian standard AS 1530.4:2014 [121] is more focused on the topic of fire. It shows how testing for fire resistance should be conducted based on the structural element. It shows where to place thermocouples while testing, as well as present the shape of the sample and the overall procedure of the test. Last, these codes only discuss either NSC or NSC and HSC with total disregard to other types of concrete such as GPC, SCC, RAC, and UHPC—all of which are very common types of concrete today.

The development of design rules that correctly represent the wide range of aggregates, cementitious materials, fibers, and the different cooling methods is very difficult to accomplish. This is because there are many variables that govern the behavior of concrete under elevated temperatures. Table 9 [5,48,75,84,97,100,108,135] shows different design rules found in the literature regarding the mechanical properties of concrete under fire. The design rules in Table 9 have been developed based on experimental results from many studies. The general trend is that the equation attempts to calculate a certain mechanical property relying on the original mechanical property at room temperature and the target temperature. However, all of these design rules are based on data from a relatively a small pool of studies. Moreover, they do not consider the variability of the aggregates in each study; in addition, they rely on developing models based on the concrete type rather than the aggregates or the cementitious materials used in each concrete mix. Although they are beneficial to a certain degree, they are still not representative of the true nature of concrete. In addition, these design rules are considered very few given the fact that engineers need to understand the mechanical properties of more than 8 concrete types. The Eurocode [5] provides equations regarding NSC for tensile strength and thermal strain only. Because there is a lack of focus on developing real models that capture the overall effects of the aggregates along with the cooling method and the different heating rates, there is a need for more data to be collected and assessed correctly to improve the current codes. This will allow civil engineers to design safer and more sustainable structures using any type of concrete and any type constituents.

**Table 9:** Equations found in the literature regarding post-fire mechanical properties of different concrete types [5,48,75,84,97,100,108,135].

Study	Equation		Concrete Type
[5]	Thermal strain:		NSC
	Siliceous aggregates:		
	ɛ_c_(ɵ) = −1.8 × 10^−4^ + 9 × 10^−6^ɵ + 2.3 × 10^−11^ɵ^3^	20 °C ≤ ɵ ≤ 700 °C	
	ɛ_c_(ɵ) = 14 × 10^−3^	700 °C ≤ ɵ ≤ 1200 °C	
	Calcareous aggregates:		
	ɛ_c_(ɵ) = −1.2 × 10^−4^ + 6 × 10^−6^ɵ + 1.4 × 10^−11^ɵ^3^	20 °C ≤ ɵ ≤ 805 °C	
	ɛ_c_(ɵ) = 12 × 10^−3^	805 °C ≤ ɵ ≤ 1200 °C	
	where ɵ is the concrete temperature (°C)		
	Tensile strength:		
	f_ck.t_(ɵ) = k_c,t_(ɵ)f_ck.t_		
	Where K depends on Figure 46 or:		
	k_c,t_(ɵ) = 1	20 °C ≤ ɵ ≤ 100 °C	
	k_c,t_(ɵ) = 1 − 1(ɵ − 100)/500	100 °C ≤ ɵ ≤ 600 °C	
[48]	Compressive strength:		NSC
	$\frac{{f^{\prime }}_{\mathrm{cr}}}{{f^{\prime }}_{c}}=1.008+\frac{T}{450\ln\left(\frac{T}{5800}\right)}\geq 0.0$	20 °C ≤ T ≤ 800 °C	
	f’_cr_ = f’_c_ (1.01 – 0.00055T)	20 °C < T ≤ 200 °C	
	f’_cr_ = f’_c_ (1.15 – 0.00125T)	200 °C ≤ T ≤ 800 °C	
	Tensile strength:		
	f’_tr_ = f’_t_ (1.05 – 0.0025T)	20 °C < T ≤ 100 °C	
	f’_tr_ = f’_t_ (0.8)	100 °C < T ≤ 200 °C	
	f’_tr_ = f’_t_ (1.02 – 0.0011T ≥ 0.0)	200 °C < T ≤ 800 °C	
	Elastic modulus:		
	$\frac{E_{\mathrm{cr}}}{E_{c}}=-0.00165T+1.033$	20 °C < T ≤ 125 °C	
	$\frac{E_{\mathrm{cr}}}{E_{c}}=\frac{1}{1.2+18{\left(0.0015T\right)}^{4.5}}$	125 °C < T ≤ 800 °C	
	E_cr_ = E_c_ (−0.00165T + 1.033)	20 °C < T ≤ 600 °C	
[75]	Compressive strength:		HWSCC
	f’_cT_ = f’_c_ (−3.062 × 10^ −9^T^3^ + 2.1085 × 10^−6^T^2^ − 3.66 × 10^−4^T + 1.02367)		
	Modulus of Elasticity:		
	E’_cT_ = E’_c_ (1.322 × 10^−11^T^4^ − 1.83 × 10^−8^T^3^ + 3.2 × 10^−6^T^2^ + 9.747 × 10^−4^T + 0.97)	20 °C ≤ T < 600 °C	
	E’_cT_ = E’_c_ (−1.53 × 10^−3^T + 1.5785)	600 °C ≤ T ≤ 900 °C	
[84]	Splitting tensile strength:		FRC
	$\frac{f_{\mathrm{rfc}}}{f_{k}\;}=\frac{1}{1+\propto {\left(T-20\right)}^{\beta }}$	25 °C *≤* T *≤* 800 °C	
		$\propto =8\times 10^{-8}$	
		β=2.41	
[97]	Compressive strength:		UHPC
	α_T,compression_ = −1.02 × 10^−3^ × T + 1.02	For20 °C ≤ T ≤ 750 °C	
	Splitting tensile strength:		
	α_T,tensile_ = −1.8 × 10^−3^ × T + 1.04	For20 °C ≤ T ≤ 200 °C	
	α_T,tensile_ = −7 × 10^−4^ × T + 0.82	For200 °C ≤ T ≤ 600 °C	
	α_T,tensile_ = −1.4 × 10^−3^ × T + 1.26	For600 °C ≤ T ≤ 750 °C	
	Elastic modulus:		
	α_T,modulus_ = 1.42 × 10^−6^ × T^2^ − 2.4 × 10^−3^ × T + 1.05	For20 °C ≤ T ≤ 750 °C	
[100]	Compressive strength:		Normal strength self-compacting concrete
	Limestone filler:		(NSCC)
	f’_cT_ = f’_c_	20 °C–100 °C	
	f’_cT_ = f’_c_ (0.87 + 0.0003T − 2.2 × 10^−6^T^2^ + 8.58 × 10^−10^T^3^)	100 °C < T ≤ 800 °C	
	Glass filler:		
	f’_cT_ = f’_c_	20 °C-100 °C	
	f’_cT_ = f’_c_ (0.922 + 0.0003T − 2.05 × 10^−6^T^2^ + 7 × 10^−10^T^3^)	100 °C < T ≤ 800 °C	
	Crushed sand filler:		
	f’_cT_ = f’_c_	20 °C-100 °C	
	f’_cT_ = f’_c_ (1.01 − 0.0008T)	100 °C < T ≤ 200 °C	
	f’_cT_ = f’_c_ (0.95 + 0.0003T − 2 × 10^−6^T^2^ + 9.5 × 10^−10^T^3^)	200 °C ≤ T ≤ 800 °C	
	Slag filler:		
	f’_cT_ = f’_c_	20 °C–100 °C	
	f’_cT_ = f’_c_ (0.93 + 0.0003T − 2.3 × 10^−6^T^2^ + 7.5 × 10^−10^T^3^)	100 °C < T ≤ 800 °C	
[100]	Compressive strength:		High strength self-compacting concrete (HSCC)
	Limestone filler:		
	f’_cT_ = f’_c_	20 °C–100 °C	
	f’_cT_ = f’_c_ (1.01–0.00031T)	100 °C < T ≤ 200 °C	
	f’_cT_ = f’_c_ (0.9 + 0.0003T–2.06 × 10^−6^T^2^ + 7 × 10^−10^T^3^)	200 °C < T ≤ 800 °C	
	Crushed sand filler:		
	f’_cT_ = f’_c_	20 °C	
	f’_cT_ = f’_c_ (0.84 – 0.00031T)	100 °C ≤ T ≤ 800 °C	
	Basalt and marble filler:		
	f’_cT_ = f’_c_	20 °C-100 °C	
	f’_cT_ = f’_c_ (1.01 – 0.0002T)	100 °C < T ≤ 200 °C	
	f’_cT_ = f’_c_ (1.019 + 10^−5^T – 1.28 × 10^−6^T^2^)	200 °C < T ≤ 800 °C	
[100]	Tensile strength:		NSCC and HSCC
	Limestone filler:		
	f_crT_ = f_ct_	20 °C–100 °C	
	f_crT_ = f_ct_ (1.06 – 0.001T)	100 °C < T ≤ 400 °C	
	f_crT_ = f_ct_ (1 – 0.0011T)	400 °C < T ≤ 800 °C	
	Crushed sand filler:		
	f_crT_ = f_ct_	20 °C	
	f_crT_ = f_ct_ (1.01 – 0.002T)	100 °C ≤ T ≤ 200 °C	
	f_crT_ = f_ct_ (0.86 – 0.0008T)	200 °C ≤ T ≤ 800 °C	
	Slag filler:		
	f_crT_ = f_ct_20 °C-100 °C		
	f_crT_ = f_ct_ (0.976 + 0.0001T–1.38 × 10^−6^T^2^)	100 °C < T ≤ 800 °C	
	Elastic modulus:		
	E_crT_ = E_c_	20 °C	
	E_crT_ = E_c_ (1.01 – 0.0015T)	100 °C ≤ T ≤ 400 °C	
	E_crT_ = E_c_ (0.78 – 0.00096T)	400 °C ≤ T ≤ 800 °C	
[108]	Compressive strength:		SCC
	f’_cT_ = f’_c_	20 °C	
	f’_cT_ = f’_c_ (0.99 – 0.002T)	100 °C ≤ T ≤ 200 °C	
	f’_cT_ = f’_c_ (0.73 – 0.0005T)	200 °C ≤ T ≤ 800 °C	
	Tensile strength:		
	f_crT_ = f_ct_	20 °C	
	f_crT_ = f_ct_ (0.99 – 0.001T)	100 °C ≤ T ≤ 800 °C	
	Elastic modulus:		
	E_crT_ = E_c_	20 °C	
	E_crT_ = E_c_ (0.84 – 0.001T)	100 °C ≤ T ≤ 800 °C	
[135]	Compressive strength:		SCC
	f’_cT_ = f’_c_ (−0.0000005T^2^ – 0.000729T + 1.01)	20 °C < T < 800 °C	
	Tensile strength:		
	f_crT_ = f_ct_ (−0.0000008T^2^ – 0.0006T + 1.06)	20 °C < T < 800 °C	
	Elastic modulus:		
	E_crT_ = E_c_ (0.0000008T^2^ – 0.00196T + 1.04)	20 °C < T < 800 °C	

There is a movement toward the development of machine learning (ML) in order to ease the solution of any civil engineering problems. One study has created a large database containing information on the frequency of identified features of selected certain RC columns under fire [136]. The database provides fire resistance analysis and spalling analysis as well as discusses the use of different ML algorithms. There are six algorithms that are most used to cover all engineering problems: Decision Trees (DT), Keras Deep Residual Neural Network (KDP), Random Forest (RF), Extreme Gradient Boosted Trees (ExGBT), TensorFlow Deep Learning (TFDL), and Light Gradient Boosted Trees (LGBT). This shows that there is an effort to summarize the effect of fire under modern methods such as ML. However, there is still a lack of review articles that can clearly capture all the data from the literature. 

**Figure 47:**
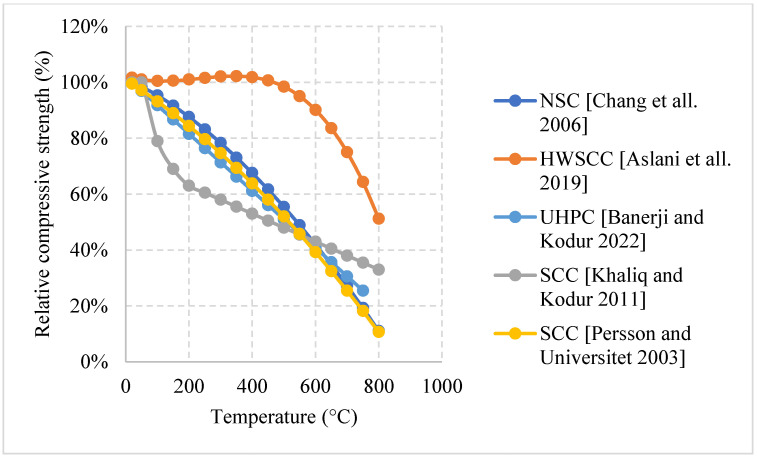
Relative compressive strength models for different types of concrete [48,75,97,108,135].

**Figure 48:**
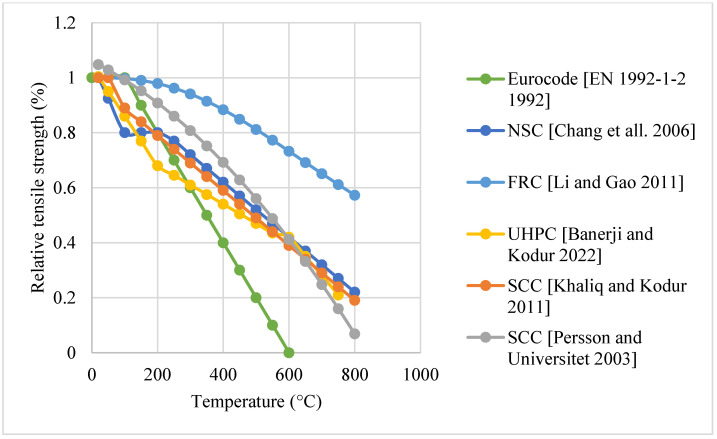
Relative tensile strength models for different types of concrete [5,48,84,97,108,135].

**Figure 49:**
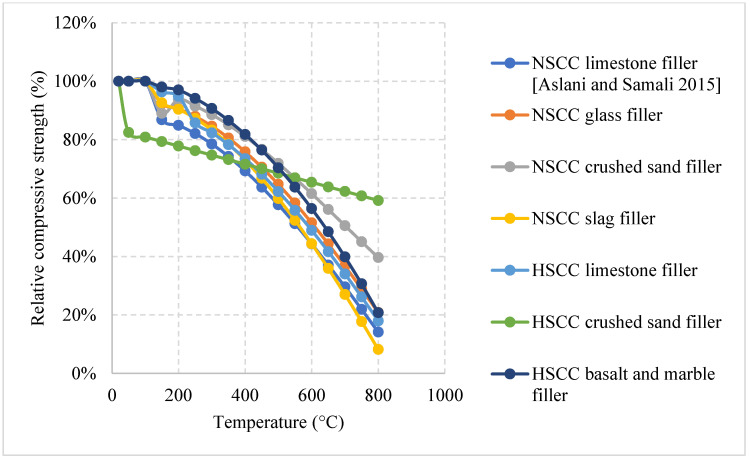
Relative compressive strength models for different types of concrete 2 [100].

**Figure 50:**
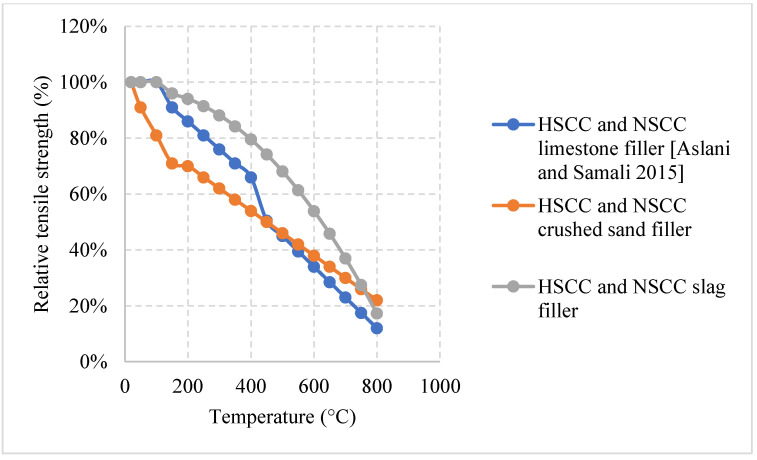
Relative tensile strength models for different types of concrete 2 [100].

**Figure 51:**
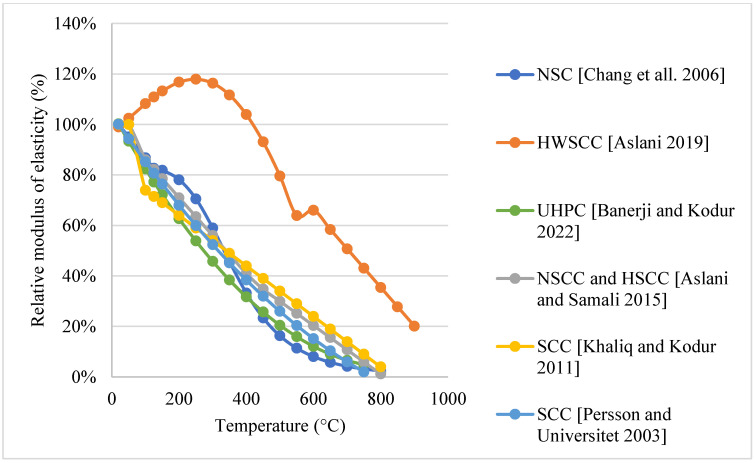
Relative modulus of elasticity models for different types of concrete [48,75,97,100,108,135].

In light of the proposed models done in the literature, it is very difficult to compare them directly because there are very few and for different types of concrete. They rely on mainly one variable (the variation in temperature) to obtain the relative strength. Moreover, they are very general. For instance, SCC can be made from different materials that can have led to extremely different behaviors. Even in Figure 47 [48,75,97,108,135], the two studies on SCC do not agree, as one of them yields a sharper decrease in strength between 20 °C and 200 °C. Furthermore, the equations yield no increase in strength of NSC, SCC, FRC, or UHPC when it was clear from the data presented in this article that there is a clear rise in strength at the early stages of heating that cannot be ignored. Figure 48 [5,48,84,97,108,135] compares the relative tensile strength of different types of concrete and it is clear that Eurocode presents an underestimate when compared to [48]. More studies should be done to verify the actual behavior of NSC. In Figure 49 and Figure 50 [100], different filler types are compared for NSCC and HSCC, and it is shown that the compressive strength behavior is generally the same. This trend was not the same when comparing the tensile strength. One clear thing to note is the behavior of slag relative to other fillers. In the compressive strength, slag was one of the worst at sustaining the strength at higher temperatures. However, it was very good in tension relative to the other fillers. All of the data presented in this study shows that there is a clear variation within the different types of concrete based on the constituents used. However, the models, as well as the codes, are still not up to date and not comprehensive of the different constituents. So, there is a need to study the effects of fire more and be able to develop/compare models to update the present standards and codes for the development of more sustainable concrete that can behave well under fire. The general trend of direct decrease is seen in Figure 51 [48,75,97,100,108,135] as well. The only exception is HWSCC in which the model predicts an increase in the strength until 300 °C. 

## 4. Performance of Advanced Materials and Construction Methods Exposed to Elevated Temperature

Recent civil engineering development aims to enhance concrete properties and to achieve durable and sustainable goals. Such advancements improve the efficiency of a wide range of applications related to structural health monitoring or the overall construction process. In addition, advancements in material science, construction materials, and methods highlight the need for research to investigate performance of these materials when exposed to elevated temperature [137,138,139]. A recent study discussed performance of cement-based sensors embedded with Multi-Walled Carbon Nanotube (MWCNT). This cement-based sensor is useful for several applications that require electrical conductivity. It was found that concrete using such material has thermal properties that range from low resistance to moderate resistance [137]. Furthermore, 3D printing is a more efficient method in many construction scenarios. This is because such a method reduces the construction time, construction waste, and labor [138]. In 3D printed concrete, the air content is reduced when compared to normal concrete. However, the overall behavior under fire was similar to that of NCC [139]. Furthermore, it was found that specimens exposed to 600 °C and 800 °C delaminated through the interface between layers due to the weak adhesion between layers [138,139]. Therefore, it is clear that there are other opportunities for research needs concerning these topics.

## 5. Conclusions

Concrete has many different types, all of which have unique mechanical properties and their performance when exposed to elevated temperature/fire; every one of these concrete types yields a unique behavior in terms of strength, cracking, spalling, and durability. For instance, NSC will not behave similar to LWC or UHPC; in addition, SCC will not yield an exact behavior to HSC or GPC. It becomes more complicated when there is a change within the concrete type itself. For instance, it is clear from the literature that varying the constituents within the concrete mix will result in a different behavior at elevated temperatures. In fact, using additional binders or air entraining agents can enhance the properties of concrete under fire. However, they could also not provide the concrete with any benefits depending on their percentages in the mix and on the other constituents they are used with. For example, adding fibers to a mix that already has silica fume can enhance the performance of concrete initially but can increase the risk of spalling later on. Moreover, applying different cooling methods is clearly another variable that affects the post-fire properties of concrete. It is shown that water cooling results in the worse outcome for any type of concrete when compared to natural cooling inside or outside the furnace. However, the researchers still favor studying the properties of concrete under natural cooling outside the furnace. Although this is not representative of the most extreme case, it is safer to study than water cooling and it can provide the engineering community with a good understanding of different constitutive behaviors under fire. Also, the codes are still underdeveloped and are not representative of concrete under fire. This is because the variety in the mixes, cooling methods, heating rates, and new materials add many challenges to address these varieties. These varieties make it difficult to conduct studies that are representative of all concrete types. Some major conclusions found in the literature are:

- Using 7% silica fume in NSC can improve its post-fire properties and yield around 60% increase in compressive strength at 300 °C. Furthermore, using air entrainers can prove to be beneficial in terms of reducing the built-up pressures within the concrete specimens.
- Adding 15% of fly ash can resist the negative effects of water cooling. However, water cooled NSC specimens will always yield worse results than naturally cooled ones.
- In lightweight concrete, using 60% fly ash as cement replacement yields the best results. A higher w/c ratio with a lower cement content will provide better results in terms of strength and cracking at every temperature. Moreover, an increase in residual strength of LWC is clear when adding fly ash or basalt furnace slag.
- Using PP fibers, plastic, or crumb rubber in higher strength concrete types such as HSC, UHPC, and FRC could reduce the risk of spalling. However, they could also reduce the overall strength of the concrete at higher percentages.
- Cracking and spalling are more significant issues in concrete that uses steel fibers. This is due to the fiber elongation and the additional stresses they have created as a consequence.
- SCC is heavily affected by the use of different binders as well as different aggregates such as limestone and can have increases in strength of up to 25%. GPC can have up to 80% increase at 400 °C due to the different reactors being implemented in the mix design and the lack of cement.
- The present models in the literature are few and still cannot be relied on. However, they do show that there is a difference in behavior of concrete. This lack of models calls for an increased focus on the topic of elevated temperatures in order to develop concrete that can behave well under fire.
- The current codes only discuss NSC and HSC using a very narrow range of aggregate types. They also neglect other types of concrete along with the effect of different cooling methods.
- Constituents such as Silica fume or Fly Ash which affect the setting time, as well as increase the long-term strength gain, have an overall positive effect on the residual properties of concrete as they provide a more compact matrix that has a higher compressive strength.

Finally, it is a fact that changing the materials in a concrete mix will change its post-fire behavior. Nonetheless, the extent of these changes is still not thoroughly investigated and more research should be done focusing on the behavior of concrete with different types of aggregates, cementitious materials, and different constituents under fire while implementing different cooling methods. 

## Figures and Tables

**Figure 46:**
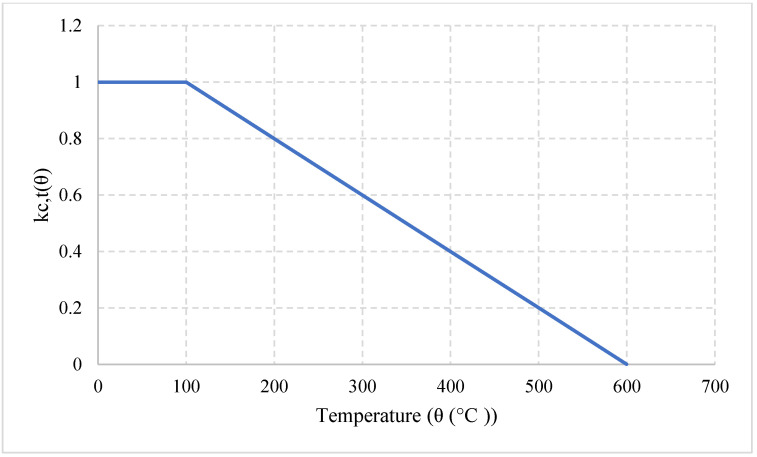
Coefficient k_c,t_(θ) allowing for decrease in tensile strength (f_ck_,t) of concrete at elevated temperatures.

**Table 1:** Important parameters of different studies on NSC [35,36,37,38,43,44,45,46,47,48,51].

Study	Cooling Type	Coarse Aggregates	Fine Aggregates	Cement	w/c Ratio	Supplementary Material	Heating Rate	Curing (Days)
[35]	natural	quartz 4/8, 8/16	quartz 0/4	OPC (42.5 MPa, rapid)	0.36	air entraining agent	10 °C/min	28
[36]	natural and water	nature granite crushed stone (maximum size of 19 mm)	natural siliceous river sand	OPC (42.5 MPa)		fly ash	10 °C/min	28
[37]	natural and water	5–20-mm pebbles	medium sand with 10% mud content	OPC	0.62	Fly ash and slag	-	28
[38]	natural	crushed Granite (10 mm and 20 mm)	river sand	OPC	0.52	-	ISO 834 standard	28
[43]	natural	granite (10 mm and 20 mm)	river sand (2 mm)	OPC (32.5 N)	0.3	-	5 °C/min	28
[44]	natural	Gravel (maximum = 155 mm)	natural sand	General purpose (GP) cement	0.45	-	NA	28
[45]	inside the furnace and water	Pakenham Blue Metal (Old Basalt) crushed (absorption = 1.2%)	lyndhurst washed fine sand (absorption = 0.5%)	OPC	0.63	Ground granulated blast furnace slag	6.25 °C/min	28
[46]	inside the furnace	Limestone, granite, and quartzite (19 mm maximum)	not applicable	OPC	0.55	-	Not specified	28
[47]	inside the furnace	10 mm maximum	sand	OPC	0.5	-	Not specified	28
[48]	inside the furnace	Siliceous aggregate	not specified	OPC	Not specified	-	1–4.5 °C/min	28
[51]	Natural 45–33	crushed gravel (19 mm maximum)	sand	OPC (52.5 N)	0.36	Silica fume and polycarboxylate-based superplasticizer	-	4

**Table 2:** Important parameters of different studies on LWC [61,62,63,64,65].

Study	Cooling Type	Coarse Aggregates	Fine Aggregates	Cement	w/c Ratio	Supplementary Material	Heating Rate	Curing (Days)
[61]	natural	basalt pumice aggregate	basalt pumice aggregate	CEM I 42.5 R	0.48, 0.21, 0.18, 0.29, and 0.25	fly ash, Basalt furnace slag	6 °C/min	7, 28, and 56
[62]	natural and water	-	Pumice (absorption = 6.38%)	CEM I 42.5	0.72, 0.74, 0.76, and 0.78	fly ash	10 °C/min	28
[63]	1 °C/min and instantly	lightweight expanded clay aggregate (4/8 mm)	lightweight expanded clay aggregate (0/2 mm)	CEM I 42.5 R	0.42, 0.45, 0.40, 0.50	silica fume, metakaolin, and liquid poly-carboxylic acid-ether superplasticizer	2 °C/min	28
[64]	natural and water	lightweight expanded clay (5 mm and 8 mm)	Dune sand	CEM I 42.5 N	0.3	micro silica	10 °C/min	28
[65]	natural and water	Not specified	Crushed and dune sand	CEM I 42.5 N	-	-	-	7 and 28

**Table 3:** Important parameters of different studies on HSC [67,68,74,75,76,77,78,79].

Study	Cooling Type	Coarse Aggregates	Fine Aggregates	Cement	w/c Ratio	Supplementary Material	Heating Rate	Curing (Days)
[67]	natural and water	-	sand and limestone < 4 mm	CEM I 42.5	0.3	silica fume	5.5 °C/min	28
[68]	natural	crushed limestone (22 mm maximum)	natural sand	CEM I 42.5 R	0.2	ground pumice and metakaolin	5 °C/min	28
[74]	natural	2/8 and 8/16 mm basalt	river sand < 2 mm	CEM I 42.5 R	0.3	polycarboxylate air-entraining agent	ISO 834	28
[75]	natural	10 mm natural magnetite	sand < 4 mm	CEM I 42.5 General Portland (GP)	0.3	fly Ash, GGBFS, and silica Fume	5 °C/min	28
[76]	natural	limestone aggregates (9.5 mm maximum)	natural fine aggregate	CEM I 42.5 R	0.3 and 0.32	silica Fume	10 °C/min	28
[77]	0.5 °C/min	crushed gravel (maximum = 19 mm)	natural sand	Brazilian cement CPV–ARI	0.32	plastic waste, rice husk, polycarboxylate and PP fibers	5 °C/min	28
[78]	inside furnace	-	natural river sand < 1 mm	CEM I 42.5 R	0.24	slag, air entraining admixture	10 °C/min	10
[79]	water and inside furnace	crushed granite (20 mm maximum)	Natural river sand	OPC	0.49	Superplasticizer of naphthalene sulphonates, silica fume and pulverized fly ash (PFA)	5–7 °C/min	90

**Table 4:** Important parameters of different studies on FRC [16,64,81,82,83,84,85,86,87,88].

Ref	Cooling Type	Coarse Aggregates	Fine Aggregates	Cement	W/C Ratio	Fiber Type	Heating Rate	Curing (Days)
[16]	Water and natural	crushed limestone	medium sand	OPC 42.5	0.26	steel fibers (30 mm) and PP fibers (20 mm)	ISO 834	58
[64]	Natural and water	lightweight expanded clay (5 mm and 8 mm)	dune sand	Type 1 OPC	0.3	micro silica and steel fibers	10 °C/min	28
[81]	Natural	4/8 mm fraction	0/4 mm fraction natural sand?	CEM I 42.5	0.43	steel fibers (13 mm), PP fibers (12 mm), and cellulose (12 mm)	10 °C/min	7
[82]	Natural	4/8 mm fraction	0/4 mm fraction natural sand?	CEM I 42.5	0.43	steel fibers (13 mm), PP fibers (12 mm), and cellulose (12 mm)	10 °C/min	7
[83]	Natural	coarse aggregate of washed siliceous nature (maximum size of 12 mm)	washed fine river sand 0/4 mm	CEM II/BL 32.5	0.5	steel (35 mm and hooked end) and PP (1 2 mm and straight)	-	28
[84]	Natural	crushed rock (maximum size of 20 mm)	natural sand	OPC	0.37, 0.30, and 0.25	steel fibers (32.6 mm) and PP fibers (19 mm)	10 °C/min	28
[85]	Natural	-	stone dust (max agg. Size = 2.3 6 mm) and crumb rubber	OPC type II	0.485	oil palm fruit fiber (30–50 mm)	5 °C/minute	28
Ongoing research	Natural and water	lightweight expanded clay (5 mm and 8 mm)	dune sand	Type 1 OPC	0.3	Micro silica and synthetic and steel fibers	10 °C/min	28
[86]	Natural (inside furnace)	dolomite aggregate (8/16 and 4/8 mm)	dolomite aggregate (0/4 mm)	CEM II/A-M(S-V) 42.5 N	0.4	PP fibers (18 mm) and hemp fibers (18 mm)	1 °C/min	28
[87]	Natural (inside furnace)	crushed limestone	dune sand	Type 1 OPC	0.5	PP fibers (15 mm)	10 °C/min	28
[88]	Water	Basalt based	quartz based natural sand	OPC	0.45	rubber (20 mm)	5 °C/min	28

**Table 5:** Important parameters of different studies on UHPC [38,47,92,93,94,95,97].

Study	Cooling Type	Coarse Aggregates	Fine Aggregates	Cement	w/c Ratio	Supplementary Material	Heating Rate	Curing (Days)
[38]	natural	Crushed gravel (19 mm maximum)	crushed fine gravel and crushed basalt (2.36 mm maximum)	CEM I-52.5 N Portland cement	0.16	silica fume, polycarboxylate-based and steel fiber	-	-
[47]	inside the furnace	-	fine bauxite aggregates	OPC	-	Steel fiber, PP fibers, and Ducorit (D4) made from cementitious mineral powder, and fine bauxite aggregates	5 °C/min and 30 °C/min	28
[92]	natural	Basalt aggregates (16 mm maximum)	machine made sand	P·II 52.5-R Portland cement	0.3	silica fume, fly ash, GGBS, steel fibers, and PP fibers	2 °C/min	56
[93]	natural	-	quartz sand and steel slag	OPC grade 42.5	0.2	silica fume, fly ash, steel fibers, and PP fibers	4 °C/min	28
[94]	inside the furnace	-	natural river sand and silica sand	CEM II 52.5 R Portland cement	0.2	silica fume, polycarboxylic type, jute fibers	0.5 °C/min	28
[95]	inside the furnace and water	-	natural river sand, pumice aggregates, and silica sand	CEM I 52.5 N Portland cement	0.2	silica fume, polycarboxylate type steel fibers, and PP fibers	1 °C/min	28
[97]	No cooling	carbonate aggregates consisting of 26A limestone	silica sand and natural sand	Type 1 cement	0.14	straight steel and PP fibers	0.5 °C/min and 2 °C/min	28 and 90

**Table 6:** Important parameters of different studies on HSC [75,101,102,103,104,105,106,107,108].

Ref.	Cooling Type	Coarse Aggregates	Fine Aggregates	Cement	w/c Ratio	Supplementary Material	Heating Rate	Curing (Days)
[75]	natural	10 mm Crushed aggregate	crushed aggregate < 4 mm	General Portland (GP)	0.3	fly Ash, GGBFS, Silica Fume, High-Range Water-Reducing Admixture (HRWRA)	5 °C/min	28
[101]	natural	crushed limestone (13 mm maximum) and expanded clay for LWSCC	siliceous quarry sand and expanded clay for LWSCC	Type 1 Portland cement	0.488	fly ash, AEA, and polycarboxylate based HRWRA	1 °C/min	28
[102]	natural	limestone aggregate or lightweight pumice for LWSCC	normal weight limestone aggregates	CEM I 42.5R	0.65, 0.6, and 0.55	oil alcohol and ammonium salt based AEA, Polycarboxylate ether HRWR, and olivine powder	5 °C/min	28
[103]	natural	crushed stone (11 mm maximum)	crushed sand	Portland cement (ASTM type II)	0.68	steel and PP fibers, fly ash, silica fume	5 °C/min	28
[104]	natural	normal coarse aggregate (density: 2.57 g/cm^3^, water absorption: 1.55%, maximum size: 15 mm)	copper slag, and normal fine aggregate (density: 2.57 g/cm^3^, water absorption: 1.77%)	Ordinary Portland cement (density: 3.16 g/cm^3^, R2O: 0.56%)	0.4	fly ash	5 °C/min	28
[105]	natural	crushed aggregates (22.5 mm maximum)	quarry sand	CEM II 32.5 R and CEM I 52.5 N	0.61 and 0.57	limestone power	1 °C/min	90
[106]	furnace	crushed limestone (16 mm maximum)	natural river sand	CEM I 42.5N	0.33–0.47	lime-stone powder (LP), basalt powder (BP) and marble powder (MP)	1 °C/min	28
[107]	Natural	Furnace slag, expanded clay, and expanded shale (10 mm)	furnace slag, expanded clay, and expanded shale (4.75 mm)	General use Portland cement	0.35, 0.36, and 0.4	fly ash, polycarboxylate ether HRWR. and silica fume	5 °C/min	28
[108]	natural	limestone (carbonate) based (10 mm maximum)	natural fine sand	Type 1 Portland cement	0.44	fly ash, slag, AEA, PP fibers, and steel fibers	5 °C/min	-

**Table 7:** Important parameters of different studies on GPC [39,43,44,117,118].

Ref.	Cooling Type	Coarse Aggregates	Fine Aggregates	Cement	w/c Ratio	Supplementary Material	Heating Rate	Curing (Days)
[39]	natural	crushed granite (10 mm and 20 mm)	river sand	-	-	sodium hydroxide, sodium silicate, and fly ash	ISO 834 standard	28
[43]	natural	gravel (10 mm and 20 mm)	river sand	-	-	metakaolin, fly ash, and alkaline activator	5 °C/min	28
[44]	natural and water	gravel (maximum = 155 mm)	natural sand	-	-	fly ash, sodium silicate solution (SSS), and sodium hydroxide solution (SHS)	NA	28
[117]	natural	gravel (5 mm and 16 mm)	River sand (2 mm)	-	-	metakaolin, fly ash, potassium silicate solution, and potassium hydroxide	5 °C/min	28
[118]	natural and water	granite rocks (10 mm)	river sand	-	-	fly ash, sodium hydroxide, and sodium silicate	5 °C/min	7

**Table 8:** Standards, codes, and technical reports on topics of fire and structures [4,5,119,120,121,122,123,124,125,126,127,128,129,130].

Organization	Country/ Region	Document Name	Document Type	Publication Date	Key Contents
American concrete institute (ACI)	USA	Code Requirements for Determining Fire Resistance of Concrete and Masonry Construction Assemblies [4]	Building code	2019	Concrete masonry, finish material and their effects on fire resistance, and clay brick and tile masonry. This document also covers concrete walls, roofs, and floors.
Eurocode (CEN)	EU	EN 1992 1-2 [5]	Building code	2004 and 2005	Details on design and construction procedures, and material properties.
American Society of Civil Engineers (ASCE)	USA	Structural Fire Protection [119]	Standard	1992	Fire safety, building design, effect of fire on wood, steel, and concrete, and the effects of fire over time.
Standards Australia	Australia	Concrete structures AS 3600:2018 [120]	Standard	2018	Structural design of different structural elements, design for fire resistance, design for durability, structural analysis,
Standards Australia	Australia	Methods for fire tests on building materials, components and structures. Part 4: Fire-resistance tests for elements of construction AS 1530.4:2014 [121]	Standard	2014	Testing procedures and specimens and failure criteria for beams, columns, ducts, control joints, floors, roofs, etc.
International Federation for Structural Concrete (fib)	EU	Fire Design of Concrete Structures –Structural Behavior and Assessment [122]	Technical Report	2008	Behavior of beams and frames, assessment of materials after fire, and repair of damaged structures.
Institution of Structural Engineers (ISE)	Britain	Appraisal of Existing Structures [123]	Technical Report	2010	Performance of existing structures before and after fire, gives classification for fire damaged structures.
Concrete Society	Britain	Assessment, Design, and Repair of Fire-Damaged Structures [124]	Technical Report	2008	Damage assessment, testing, repair methods, and effects of fire on construction materials.
Fire Safety Journal/CIB W14	N/A	The Repairability of Fire-Damaged Structures [125]	Journal Article/Technical Report	1990	Assessment, classification, and reparability of fire damaged concrete structures.
Cement Concrete & Aggregates Australia	Australia	Fire Safety of Concrete Buildings [126]	Building code	2010	Effects of fire on concrete members, a framework for regulations, and assessment of fire damaged structures.
National Fire Protection Association (NFPA)	USA	NFPA 5000: Building Construction and Safety Code [127]	Building code	2002	Specifications on different building materials, discussion on fire-retardant-treated wood, and fire security systems.
Concrete Reinforcing Steel Institute (CRSI)	USA	Fire Resistance of Reinforced Concrete Buildings (ETN-B-1-16) [128]	Technical Report	2016	Concrete covers and design of concrete to resist fire.
Canadian Commission on Building and Fire Codes (CCBFC)	Canada	National Fire Code of Canada 2015 [129]	Building code	2015	Technical provisions related to construction, and the design and construction of specific building elements.
International Organization for Standardization (ISO)	International	ISO/TS 16733-2:2021 Fire safety engineering—Selection of design fire scenarios and design fires [130]	Standard	2021	Specifications for fire design, and procedures for selecting of design fire scenarios.
